# Continuous *N*-alkylation reactions of amino alcohols using γ-Al_2_O_3_ and supercritical CO_2_: unexpected formation of cyclic ureas and urethanes by reaction with CO_2_

**DOI:** 10.3762/bjoc.13.36

**Published:** 2017-02-21

**Authors:** Emilia S Streng, Darren S Lee, Michael W George, Martyn Poliakoff

**Affiliations:** 1 School of Chemistry, University of Nottingham, University Park, Nottingham, NG7 2RD, UK; 2 Department of Chemical and Environmental Engineering, University of Nottingham Ningbo China, 199 Taikang East Road, Ningbo 315100, China

**Keywords:** continuous flow, heterocycle, *N*-alkylation, self-optimisation, supercritical CO_2_

## Abstract

The use of γ-Al_2_O_3_ as a heterogeneous catalyst in scCO_2_ has been successfully applied to the amination of alcohols for the synthesis of *N*-alkylated heterocycles. The optimal reaction conditions (temperature and substrate flow rate) were determined using an automated self-optimising reactor, resulting in moderate to high yields of the target products. Carrying out the reaction in scCO_2_ was shown to be beneficial, as higher yields were obtained in the presence of CO_2_ than in its absence. A surprising discovery is that, in addition to cyclic amines, cyclic ureas and urethanes could be synthesised by incorporation of CO_2_ from the supercritical solvent into the product.

## Introduction

*N*-alkylated amines are an important motif present in a range of pharmaceutically and industrially useful chemicals; the alkylation of amines is a commonly used reaction in process R&D toward the synthesis of drug candidates [1–3]. Traditional methods to produce such compounds frequently employ toxic alkylating agents or harsh reagents that can generate stoichiometric quantities of waste, e.g., boron salts from reductive amination [4]. Hydrogenation offers a greener approach but is often only applicable to simple substrates due to chemoselectivity issues. An approach that has received much attention recently is the concept of hydrogen borrowing catalysis [5–19]. The coupling of alcohols and amines is made possible by the catalysts ability to take two H atoms from the alcohol, oxidising it to an aldehyde. The aldehyde then reacts with the amine affording an imine, which is subsequently reduced by transferring two H atoms back from the catalyst. In this case the only byproduct is water. Another approach to *N*-alkylation in which water is the only byproduct is the direct substitution of alcohols with amines. It is an attractive method; however, it requires significant activation of the alcohol or amine to proceed efficiently, and often a heterogeneous catalyst at elevated temperature and/or pressure is employed [20–28]. As these reactions are mostly carried out in high pressure systems, they are particularly suitable for the use of supercritical solvents. Supercritical solvents are highly compressed and/or heated gases that are beyond the critical point (e.g., the critical point for CO_2_ is 31.1 °C and 73.9 bar); in this phase the gas exhibits unique properties and behaves both like a liquid and gas. Using inert supercritical gases as reaction solvents is a greener alternative to using conventional flammable or toxic solvents; furthermore post-reaction separation is simplified as the gas/liquid phases separate upon cooling. The use of supercritical methanol (scMeOH) for *N*-alkylation reactions has been reported before [29–30].

Our own investigations with heterogeneous catalysis in supercritical carbon dioxide (scCO_2_) have mainly been focused on continuous flow systems and the etherification of alcohols, where alcohols are activated by heterogeneous catalysts [31–38]. We have usually employed γ-alumina as the catalyst, as this is a simple, readily available and environmentally benign catalyst that is often overlooked and it is used merely as a support for other catalysts [39–43]. The use of γ-alumina for the methylation of aniline with dimethyl carbonate has been reported [44]. In this paper, we chose to study the intramolecular and intermolecular alkylation of amino alcohols using γ-Al_2_O_3_ with scCO_2_ as the solvent and employed self-optimisation [45–46] to explore the defined parameter space to effectively identify the highest yielding and optimal conditions in a relatively short timeframe.

## Results and Discussion

**Scheme 1:**
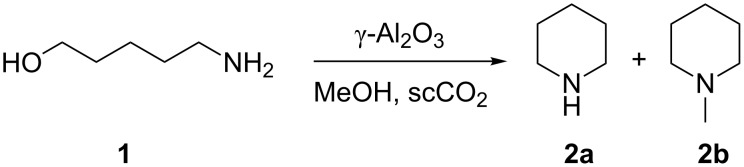
Target reaction – intramolecular cyclisation of **1** followed by *N*-methylation with methanol to yield **2b**.

**Figure 1:**
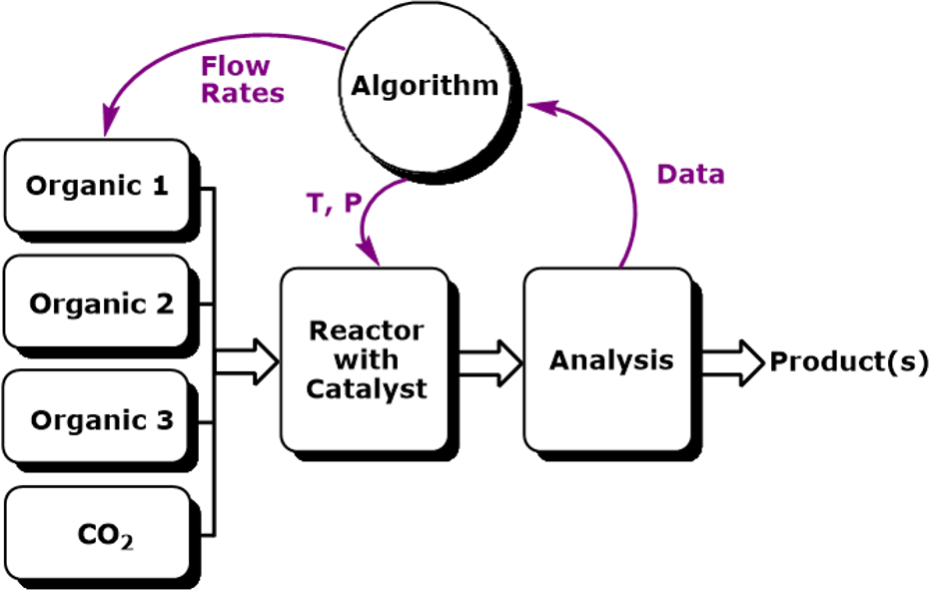
Simplified schematic demonstrating a self-optimising reactor [34–35,37,44]. The reagents are pumped into the system where they are mixed and then flowed through a reactor filled with catalyst. The output of the reactor is analysed by an on-line GC. The response (e.g., yield) of this analysis is then sent to an optimising search algorithm (e.g., SNOBFIT), which then changes the conditions (e.g., flow rates and temperature) in order to maximise the response of the analysis.

To investigate our hypothesis that γ-Al_2_O_3_ with scCO_2_ could be successfully applied to the amination of alcohols, we chose to employ a self-optimising reactor (Figure 1, see Supporting Information File 1 for details) to streamline the optimisation process using 5-amino-1-pentanol (**1**) as the model substrate and methanol as the alkylating agent (Scheme 1). For this reaction, self-optimisation is important as multiple products were identified that could form in parallel; from **1** the possible products we expected to see were a mixture of piperidine (**2a**), *N*-methylpiperidine (**2b**), *N*- and *O*-methylated **1**, as well as oligomers. We chose to target **2b** only for self-optimisation.

We targeted *N*-methylpiperidine (**2b**) using the self-optimisation approach with SNOBFIT as the optimising algorithm [47] and GC analysis as the analytical tool providing the responses for the self-optimisation. This methodology allows high yielding conditions to be found, minimising the formation of byproducts. The temperature and the flow rate of the reaction were optimised in both the presence and absence of scCO_2_ (Figure 1).

**Figure 2:**
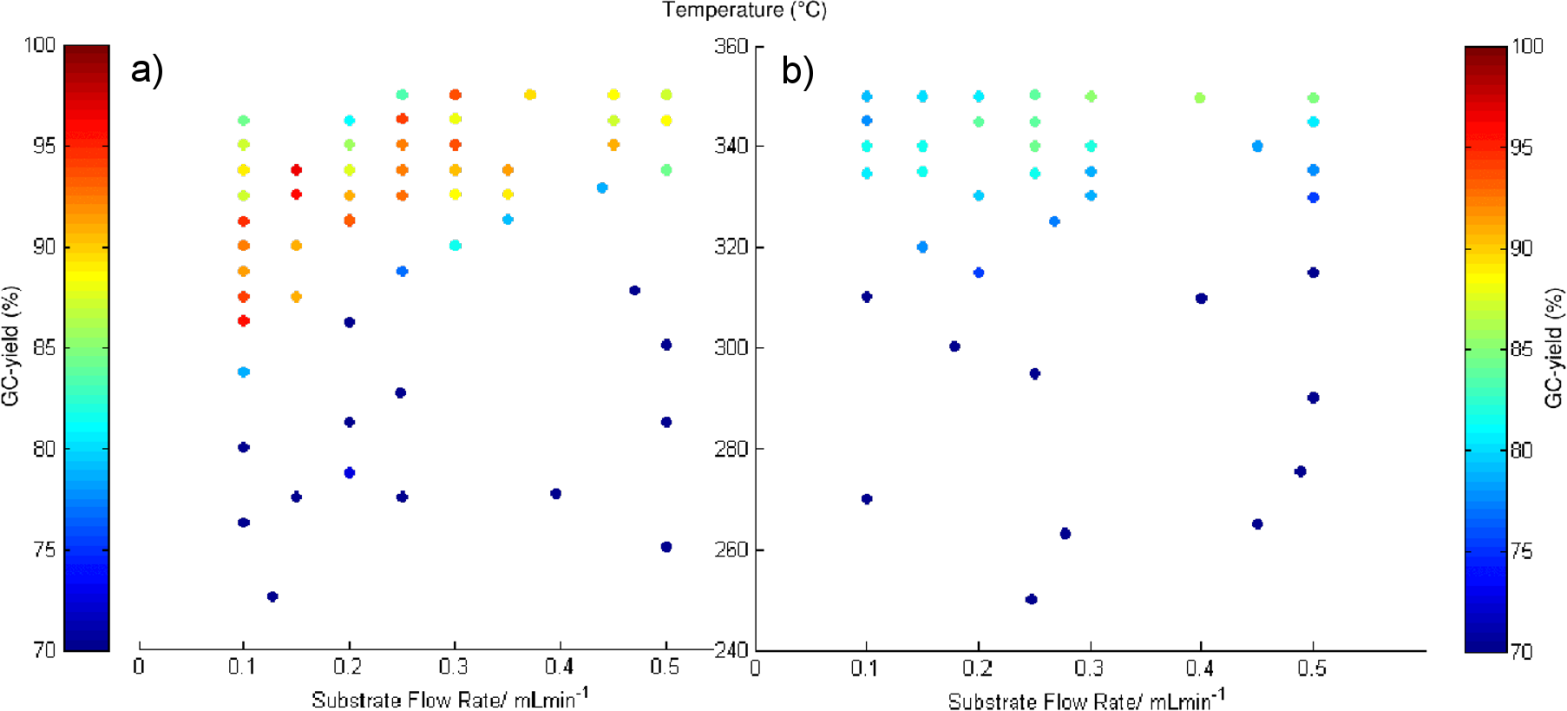
Result of the SNOBFIT optimisation for *N*-methylpiperidine (**2b**) with and without CO_2_ showing yields ≥70%. Figure a (left) shows the yields for the experiment carried out in scCO_2_ at different temperatures and flow rates; Figure b (right) shows the results without CO_2_. Conditions: Temperature 250–350 °C, substrate flow (0.5 M solution in MeOH) 0.1–0.5 mL min^−1^, 100 bar, when applicable 0.5 mL min^−1^ CO_2_.

The results of the optimisations are shown in Figure 2, and the conditions with the highest yields of **2b** are shown in Table 1. During these experiments the parameter space was extensively studied and high yields were achieved at several different conditions. This provides confidence that our optimal yield was the global optimum within the studied limits of the reaction. It can be seen from Figure 2 that, when the reaction was carried out in scCO_2_, high yields (up to 96%) for **2b** were achieved (Figure 2a, Table 1, entries 1–3). In the absence of scCO_2_ the percentage yield was good but the highest yields were ca. 8–11% less (Figure 2b, Table 1, entries 4–6) compared to when scCO_2_ was present. Clearly scCO_2_ is beneficial as a solvent in the formation of **2b**.

**Table 1:** The highest yields of **2b** found by the optimisations carried out with CO_2_ (entries 1–3) and without CO_2_ (entries 4–6).^a^

Entry	*T* (°C)	Flow rate (mL min^−1^)	Yield **2b** (%)^b^

1^c^	340	0.3	94
2^c^	310	0.1	94
3^c^	330	0.15	96
4^d^	350	0.4	86
5^d^	350	0.3	85
6^d^	350	0.5	83

^a^0.5 M solution of **1** in MeOH, 100 bar system pressure. ^b^Yields based on GC analysis. ^c^With 0.5 mL min^−1^ CO_2_. ^d^No CO_2_ used.

The optimal region for synthesising **2b** turned out to be quite broad, as high yields were obtained at a variety of conditions. At lower flow rates (0.1 mL min^–1^) and hence longer residence times, yields of 94% were observed at 310 °C (Table 1, entry 2). Increasing the temperature by 30 °C led to an increase in the rate of cyclisation and methylation which then allowed for faster flow rates to be used under this operating temperature whilst still maintaining the same yield of **2b** (Table 1, entry 1). Hence, three times the amount of material could be processed in the same time using this elevated temperature, i.e., higher productivity.

After optimisation with the model substrate **1** in methanol, the application of these reaction conditions to a small range of different alcohols was studied. Initially we repeated the model reaction to demonstrate that the approach is repeatable and that the conditions found during the optimisation were indeed the optimum (N.B. We chose the conditions that afforded the highest yield). Pleasingly, full conversion of **1** was obtained and an identical yield of **2b** was observed (Table 2, entry 1). After showing that the conditions were repeatable, we applied them to several different alcohols by flowing a starting mixture of **1** with the alcohol as the solvent (Table 2, entries 2–4). As might be expected, the cyclisation to *N*-alkylated piperidines was observed for the primary alcohols. The yield of the corresponding *N*-alkylated piperidine falls as the longer chain alcohols are reacted. When the secondary alcohol isopropanol was used as the solvent, no *N*-alkylation was observed and piperidine **2a** was found as the major product. As this catalyst system has been used previously for the etherification of alcohols [31–38], it is possible that ethers of the alcohols could be formed. In the case of **2d,** dibutyl ether was the major byproduct, but in most other cases only small amounts of the corresponding ethers were observed. When the reaction with isopropanol was repeated without scCO_2_ the same selectivity was observed. However, when primary alcohols were run in the absence of scCO_2_ the yields of the corresponding *N*-alkylated products were lower and more piperidine **2a** was observed. These results suggest that the rate of intermolecular alkylation is faster in scCO_2_, while the rate of intramolecular cyclisation is not significantly affected by the presence of scCO_2_ and thus proceeds faster than the intermolecular reaction.

**Table 2:** Cyclisation and *N*-alkylation of **1** with different alcohols.^a^

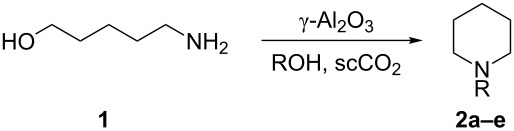		

Entry	R =	Yield (%)^b,c^

1	Me **2b**	94%
2	Et **2c**	82%
3	*n*-Bu **2d**	73%
4	iPr **2e**	0% (**2a** 80%)

^a^Conditions: **1** (0.5 M in ROH), 340 °C, substrate flow: 0.3 mL min^−1^, CO_2_ flow: 0.5 mL min^−1^, 100 bar.; ^b^Determined by GC analysis of the reaction mixture. ^c^The remaining materials are unidentified side products.

**Scheme 2:**
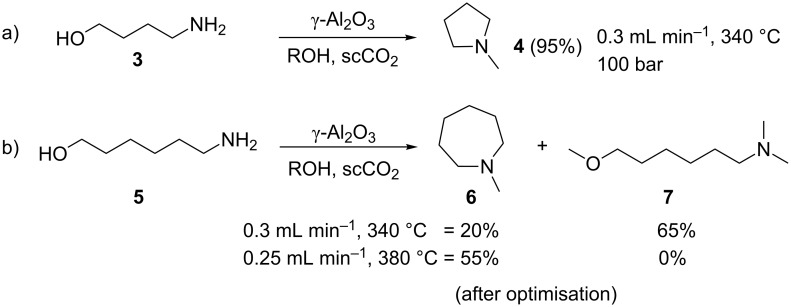
Cyclisation and *N*-alkylation of 1,4- and 1,6-amino alcohols.

We also explored the cyclisation and *N*-alkylation of different amino alcohol substrates. Initially we investigated the effect of simply changing the alkane chain length. Starting with 4-amino-1-butanol (**3**) under the model conditions afforded the desired *N*-methylpyrrolidine (**4**) in 95% yield. Extending the alkyl chain using 6-amino-1-hexanol (**5**), however, favoured methylation over intramolecular cyclisation as only 20% of the cyclised product **6** was observed. The major product was 6-(dimethylamino)-1-methoxyhexane (**7**, Scheme 2), which was formed by both *O*- and *N*-methylation of the starting material. Self-optimisation of the reaction of this substrate was performed in order to try and locate the optimal conditions for the highest yield of **6**. Within the parameters explored, it was found that higher reaction temperatures increased the selectivity and yield of **6** up to 55%. This relatively modest yield could not be optimised further.

Ethanolamine **8** was used to explore the potential competition between the intra- and intermolecular etherification and amination. In this case we observed no azridine or *N*-methylaziridine, which would be expected from the intramolecular closure of **8**, consistent with the results observed with bromoalkylamines [48], and suggesting the rate of closure for three-membered rings is slower than that of five- and six-membered rings. We cannot rule out the formation of aziridine as an intermediate in the formation of the dimeric products that were observed. The reaction with ethanolamine yielded three products (Table 3), *N*-methylmorpholine (**9**), 1,4-dimethylpiperazine (**10**) and the fully *N*- and *O*-methylated ethanolamine **11**. Under the standard conditions, **11** was the major product, and as the temperature was increased, the amount of **10** increased. When the parameter space was explored using the self-optimisation approach the selectivity to **10** was increased to 63%. The etherification/deamination pathway forming **9** could not be optimised above 11% as the dehydration or methylated products were present as the major products in all cases. These results prompted us to explore the use of more functionalised amino alcohols in an attempt to access these heterocycles more cleanly and to allow us to further examine the deamination reactivity that produces **9**.

**Table 3:** Reactions of ethanolamine.^a^

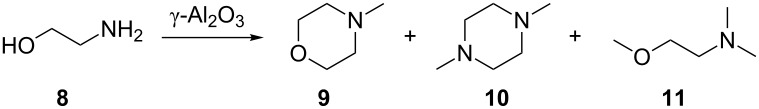						

Entry	Flow rate (mL min^−1^)	Temperature (°C)	Conversion (%)	Selectivity (%)^b^		
				**9**	**10**	**11**

1^a^	0.3	340	100	<1	13	72
2^c^	0.1	370	100	11	48	0
3^c,d^	0.1	360	100	5	63	3

^a^Conditions: **8** 0.5 M (or 1.0 M) solution in MeOH, 0.5 mL min^−1^ CO_2_, 100 bar; ^b^Based on GC analysis of the reaction mixture, remaining material is a mixture of unidentified side products; ^c^Substrate 1.0 M solution in MeOH; ^d^After self-optimisation had been run targeting high yield of **10**.

Diethanolamine **12** is expected to produce a cleaner cyclisation pathway to *N*-methylmorpholine (**9**) via intramolecular etherification. When diethanolamine **12** in methanol was reacted using the standard conditions (Table 1, entry 1), *N*-methylmorpholine (**9**) was obtained but only in 24% yield; however, when the conditions were changed in an attempt to optimise the yield, it became apparent that the reactivity of **12** was more complicated. Running the reaction at 380 °C and 0.3 mL min^−1^ resulted in 46% of **9** being obtained but, at lower temperatures, different products were obtained. For example, when the reaction was run at 250 °C (Table 4, entry 1), oxazolidinone **13** was observed as the major product (52%) together with **14**, a dimer of the starting material **12** as the main byproduct (42%).

**Table 4:** Showing the effect of conditions on the reaction of diethanolamine **12** to form carbamate **13** and piperazine **14**.^a^

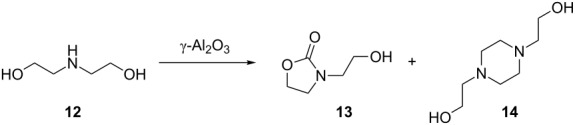							

Entry	Conc. (M)	*T* (°C)	*P* (bar)	Flow rate (mL min^−1^)	Conv. (%)^b^	Selectivity (%)^b^	
						**13**	**14**

1	0.5	250	100	0.3	53	52	42
2	0.5	250	100	0.2	98	20	65
3	0.5	250	100	0.1	100	0	61^c^
4	0.5	240	100	0.3	48	69	26
5	0.2	250	100	0.3	80	42	38
6	0.2	250	150	0.3	73	65	19
7	1.0	250	150	0.2	56	73	22
8	1.0	275	100	0.2	100	8	63^d^

^a^**12** in ethanol, 0.5 mL min^−1^ CO_2_. ^b^Based on GC analysis of the reaction mixture. ^c^12% of mono-*O*-ethylated **14**. ^d^Trace of mono- and bis-ethylated **14**.

Formation of **13** involves incorporation of the CO_2_ solvent into the product. Despite the very large number of reactions studied in scCO_2_, there are relatively few examples of incorporation of CO_2_ into the product. In this case, incorporation presumably occurs via the formation of a carbamate intermediate. This surprising formation of **13** suggests the incorporation of CO_2_ into **12** with the dimer formation as a competing reaction. In fact, when further conditions were studied, it became apparent that the dimer **14** could be formed from oxazolidinone **13** as increasing the residence time led to an increase in selectivity of **14** over **13** (Table 4, entry 2). Indeed, when **13** was used as the starting material, the major product that was isolated was **14**; and this reactivity of **13** has been reported previously in batch reactions [49]. Increasing the residence time further (Table 4, entry 3) resulted in the oxazolidinone **13** not being detected and **14** was the major product together with a small quantity of mono *O*-ethylated **14**. Reducing the temperature gave a better selectivity to the oxazolidinone **13** (Table 4, entry 4) and lowering the concentration, increased the conversion but gave a poor selectivity (Table 4, entry 5). Increasing the pressure to 150 bar had a positive effect on the selectivity toward **13** (Table 4, entry 6) and increasing the concentration of **12** to 1 M gave the highest selectivity for **13** (Table 4, entry 7). Further increasing the temperature to 275 °C only served to increase the selectivity towards **14** (Table 4, entry 8). From these conditions, it appears that the incorporation of CO_2_ is fast but the rate of conversion to **14** is dependent on the pressure of the system, the temperature of the reactor, the residence time and to some extent the concentration of the amino alcohol in the alcohol. A higher pressure of CO_2_ appears to slow the rate of conversion of **13** to **14**, whilst elevated temperatures appear to accelerate the rate. Increasing the residence time allows more time for **13** to be converted into **14** and hence the higher selectivity for it and the appearance of trace amounts of mono- and bis-ethylated **14**.

**Scheme 3:**
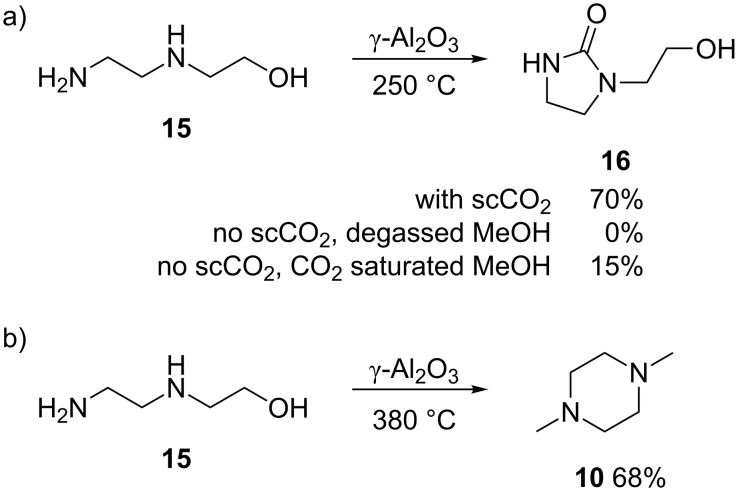
a) Reactions highlighting the incorporation of CO_2_ in to **16**. b) High temperature reaction of **15** yielding *N*,*N’*-dimethylpiperazine (**10**).

We have studied the incorporation of CO_2_ further by investigating the reaction of *N*-(2-aminoethyl)ethanolamine **15**. The use of **15** as a starting material might be expected to produce high selectivity for the corresponding imidazolidinone **16** via the incorporation of CO_2_. The competing oxazolidinone formation should be limited as the nucleophilicity of nitrogen is more than that of the oxygen. Furthermore, the formation of dimers were expected to be supressed as **16** does not contain a “CO_2_ unit” that can serve as a leaving group. This was indeed the case as, at 250 °C, 85% selectivity, 70% yield for **16** was observed when the reaction was run in scCO_2_ (Scheme 3a). In the absence of CO_2_ as a solvent the formation of imidazolidinone **16** was not observed. When the starting solution was pre-saturated with CO_2_ and run in the absence of CO_2_ as a solvent, **16** was formed in 62% selectivity, 15% yield from 24% conversion of the starting material. This poor conversion suggests that CO_2_ is needed in an excess for the reaction to be successful, and the use of CO_2_ as the solvent as well as a reagent in this case provides the highest possible concentration of CO_2_. To establish whether any dimers are formed when **16** is exposed to the catalyst bed for an extended time or to higher reaction temperatures, a solution of **16** in iPrOH (0.5 M) was flowed at 250 and 275 °C, but no dimers were detected and unreacted **16** was the main product observed. The reaction of **15** with CO_2_ could be supressed using higher temperatures, for example at 380 °C in methanol the intramolecular cyclisation is favoured and *N*,*N’*-dimethylpiperazine (**10**) is obtained as the major product in 68% yield (Scheme 3b, 380 °C at 1 mL min^−1^), and no imidazolidinone **16** was detected.

## Conclusion

**Scheme 4:**
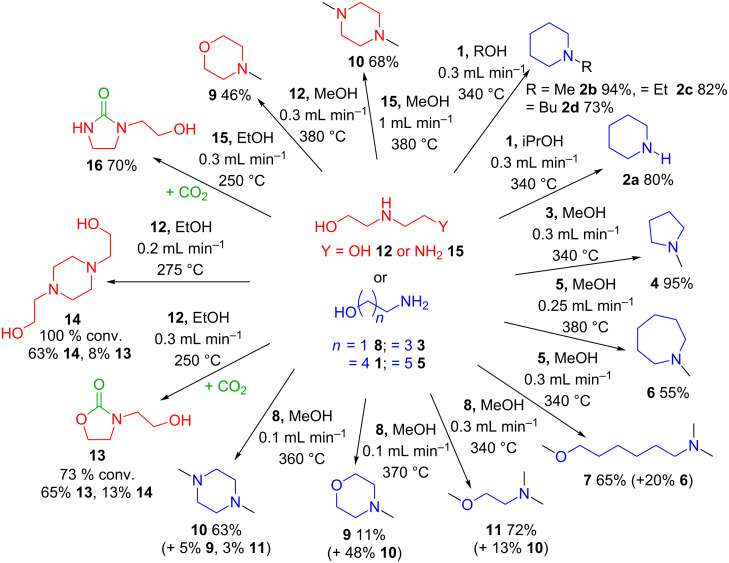
Summary of products obtained from the reactions of amino alcohols over γ-Al_2_O_3_ in scCO_2_.

Using a self-optimising reactor and a simple heterogeneous catalyst, γ-Al_2_O_3_, moderate to high yields of several alkylated cyclic amines, formed in a two-step intramolecular cyclisation/*N*-alkyation reaction, using amino alcohols and simple alcohols has been achieved (Scheme 4).

Using scCO_2_ as the solvent proved to be beneficial to the yield of cyclic *N*-alkylated amines, in particular for the *N*-alkylation step which was arrested in the absence of scCO_2_. The intramolecular cyclisation of the amino alcohols was favoured at higher temperatures in both the presence and absence of scCO_2_. Increasing the primary alcohol length led to slightly lower yields of the target products whereas secondary alcohols did not react with the amines at all. Varying the chain length of the amino alcohol produced the corresponding *N*-alkylated five- (**4**) and seven-membered ring (**6**), three-membered aziridine rings were not detected. Competing *N*- and *O*-alkylation was observed at higher temperatures with ethanolamine (**8**) and 6-amino-1-hexanol (**5**), suggesting ring closure is slower in these cases. Ethanolamine (**8**) produced dimers as the major products, mainly via the amination pathway; however, some esterification/deamination was observed as *N*-methylmorpholine (**9**) was also detected. CO_2_ incorporation in **12** and **15** was perhaps the most surprising result as this occurred at lower temperatures compared to the cyclisation, however at higher temperatures intramolecular reactions were favoured. The formation of oxazolidinones was shown to be reversible releasing CO_2_ as dimers are formed. Imidazolidinones were shown to be stable to further reaction and no release of CO_2_ was observed under the conditions studied. Further optimisation and investigations into the incorporation of CO_2_ are in progress.

## Experimental

**Figure 3:**
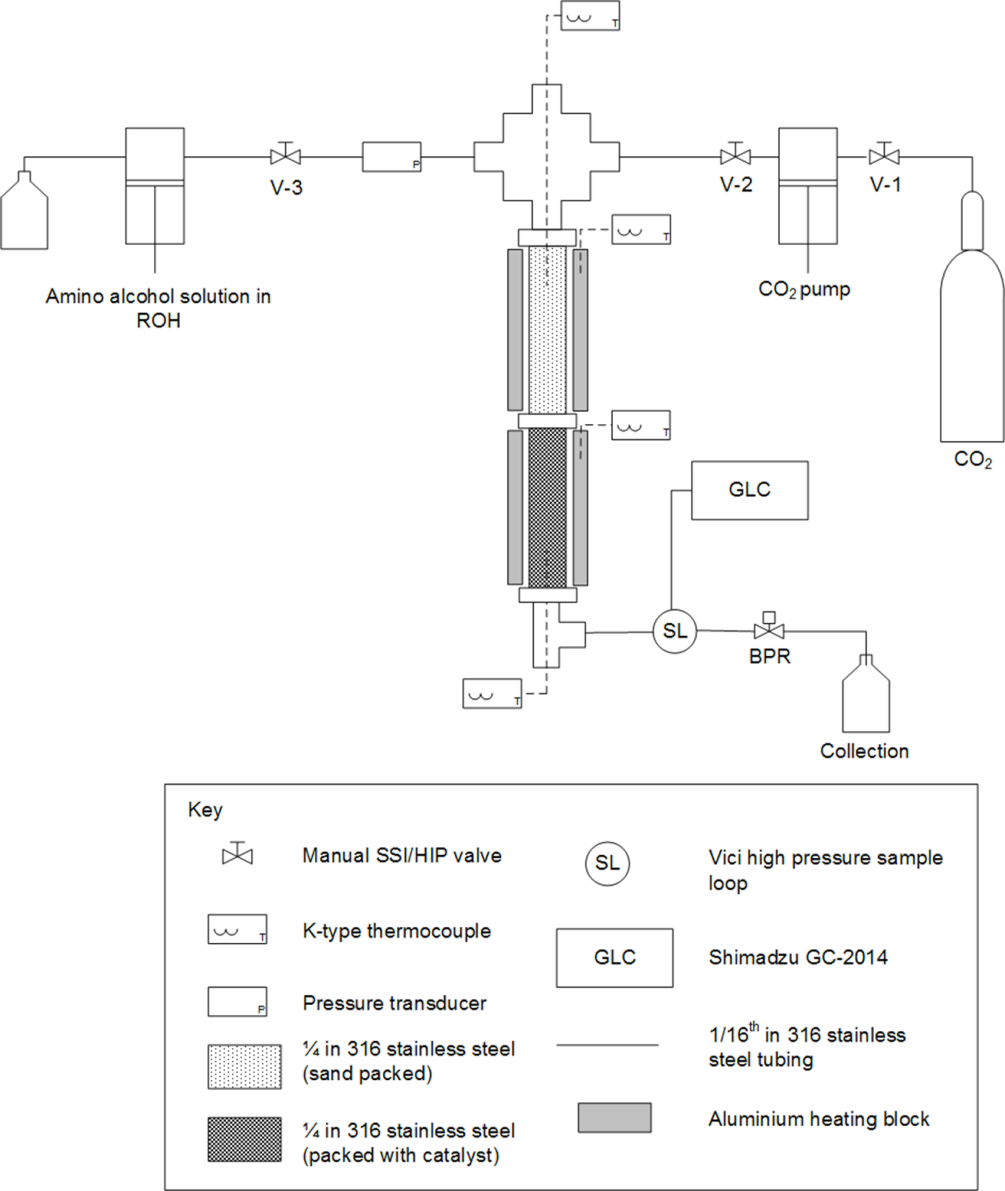
Diagram of the high pressure equipment used in the experiments.

CAUTION! The described reactions involve high pressures and require equipment (Figure 3) with appropriate pressure ratings.

All reagents and solvents were purchased from commercial sources and used as received. CO_2_ was supplied by BOC Gases (99.8%). The γ-alumina (PURALOX NWa155) was supplied by SASOL. It was sieved before use, to obtain the desired particle size (125–170 μm), which was used as the catalyst. Reaction mixtures were analysed using GC, GC–MS, ^1^H and ^13^C NMR. Compounds **1a–c**, **4**, **9**, **10**, **13**, **14**, **16** were obtained from Aldrich and used as standards. **1d,e** [50], **6** [51], **7** [52], and **11** [53] were identified as previously described in the literature.

GC analysis was carried out using the following instrument and conditions: Online Shimadzu GC-2014 with a high pressure sample loop and an OPTIMA delta-3 column (30 m, 0.25 mm ID, 0.25 µm FT): hold 50 °C 4 min, ramp to 100 °C at 25 °C/min, ramp to 250 °C at 10 °C/min, hold for 2 min, pressure 132.1 kPa, purge 3.0 mL/min split ratio 40.

The high pressure continuous set-up (Figure 3) employed in the described reactions consisted of a HPLC pump through which a solution of the desired amino alcohol in an alcoholic solvent was delivered. A stainless steel reactor (1/4’’ tube, 1.83 mL volume) was packed with γ-alumina (approx. 2 g) and attached below a pre-heater column (1/4’’ tube, 1.83 mL volume) that was packed with sand to increase mixing. A crosspiece was used to mix the CO_2_ and reagent flows before the reactors and the resulting product mixture was collected downstream of the back pressure regulator. The sampling to the on-line GC was done with a high pressure sample loop (Vici, 0.5 μL), which allowed a sample to be taken from the reaction flow. During optimisations a sample was taken once the conditions had been changed and stable state had been reached (10 min).

Some experiments were carried out by using a self-optimising reactor which has been described in detail previously [34–35,37]. All SNOBFIT [47] optimisations were performed within the following limits: Temperature 250–380 °C and flow rate 0.1–1.0 mL min^−1^. The number of points produced by each call to SNOBFIT (n_req_) was 6, and 10% of all the points were requested as global points (p = 0.1). The results at each condition were determined by GC analysis (programme time 20–23 min) and the pressure of the system was controlled by a back-pressure regulator at the outlet and was adjusted manually.

## Supporting Information

File 1 Experimental data.
