# Supplementary material for: Continuous N-alkylation reactions of amino alcohols using γ-Al2O3 and supercritical CO2: unexpected formation of cyclic ureas and urethanes by reaction with CO2
Source: Beilstein J Org Chem. 2017 Feb 21;13:329–37. doi: 10.3762/bjoc.13.36 (PMC5331277; doi:10.3762/bjoc.13.36)
Supplement: File 1 — Experimental data. [file Beilstein_J_Org_Chem-13-329-s001.pdf]

Supporting Information

for

Continuous *N*-alkylation reactions of amino alcohols using γ-Al_2O_3 and supercritical CO_2: unexpected formation of cyclic ureas and urethanes by reaction with CO_2

Emilia S. Streng[1], Darren S. Lee[1], Michael W. George*[1,2] and Martyn Poliakoff*[1]

Address: [1]School of Chemistry, University of Nottingham, University Park, Nottingham, NG7 2RD, UK and [2]Department of Chemical and Environmental Engineering, University of Nottingham Ningbo China, 199 Taikang East Road, Ningbo 315100, China

Email: Michael W. George - mike.george@nottingham.ac.uk; Martyn Poliakoff - martyn.poliakoff@nottingham.ac.uk,

*Corresponding author

Experimental data

Contents

1. Optimisation results S2
2. NMR characterisation data S10
3. GC traces of reaction mixtures S21
4. References S31

1. Optimisation results

All the described optimisation experiments were carried out using the SNOBFIT[1] algorithm. The number of points produced by each call to SNOBFIT (n_{req}) was 6, and 10% of all the points were requested as global points (p = 0.1). The results at each condition were determined by GLC-analysis. The substrate was either a 0.5 M or a 1.0 M solution in MeOH. All the points produced for the reported optimisations are listed in Tables S1–S7. The optimal point (region) was verified by repeating the reaction at those conditions, and injecting the resulting mixture into the GC. In some cases, the obtained values were lower than that measured during the optimisation, and so the reported optimal yields were the ones calculated from the injections.

Table S1. The conditions during the optimisation for 2b in the presence of CO_2.[a]

Measurement no.	Temperature (°C)	Flow Rate (mL/min)	GC-yield of **2b** (%)
1	250	0.13	0.0
2	270	0.40	29.9
3	291	0.25	40.2
4	331	0.44	33.8
5	271	0.40	78.5
6	350	0.37	89.4
7	311	0.47	86.9
8	340	0.1	92.5
9	320	0.1	24.1
10	300	0.5	6.0
11	285	0.5	58.2
12	280	0.1	0.0
13	260	0.5	78.6
14	295	0.1	64.6
15	305	0.2	90.9
16	310	0.15	94.4
17	325	0.1	90.8
18	340	0.45	84.4
19	345	0.1	83.2
20	350	0.25	87.3
21	350	0.5	88.3
22	350	0.45	92.7
23	330	0.25	96.2
24	330	0.15	91.2
25	315	0.1	72.8
26	275	0.2	29.7
27	270	0.25	94.3
28	310	0.1	93.8
29	325	0.2	87.0
30	330	0.1	96.3
31	335	0.15	85.9
32	340	0.2	92.1
33	340	0.25	88.6
34	345	0.5	87.0
35	345	0.45	92.1
36	335	0.25	88.9
37	335	0.1	81.4
38	320	0.3	76.6
39	315	0.25	95.8

40	305	0.1	91.0
41	320	0.15	78.9
42	325	0.35	87.8
43	335	0.2	84.2
44	335	0.5	93.4
45	340	0.3	93.7
46	350	0.3	88.0
47	345	0.3	94.0
48	345	0.25	89.1
49	330	0.35	88.6
50	330	0.3	93.1
51	270	0.15	0.0
52	265	0.1	0.0
53	285	0.2	49.7
54	330	0.2	90.9
55	335	0.3	90.6
56	335	0.35	91.1
57	345	0.2	80.9

a) Conditions: 250–350 °C and 0.1-0.5 mL/min (0.5 M of **1** in MeOH), 100 bar, 0.5 mL/min CO_2.

Table S2. The points measured during the optimisation for product 2b in the absence of CO_2.[a]

Measurement no	Temperature (°C)	Flow Rate (mL/min)	GC-yield of **2b** (%)
1	250	0.25	0.0
2	263	0.28	0.0
3	275	0.49	2.3
4	300	0.18	62.7
5	325	0.27	77.1
6	350	0.40	85.7
7	340	0.10	81.3
8	335	0.50	77.8
9	315	0.50	54.5
10	310	0.10	69.8
11	290	0.50	10.3
12	270	0.10	25.7
13	315	0.20	75.5
14	335	0.25	81.5
15	340	0.15	81.6
16	340	0.45	78.0
17	350	0.10	79.1
18	350	0.25	83.7
19	350	0.30	85.2
20	345	0.25	83.6
21	345	0.20	83.6
22	335	0.10	80.7
23	330	0.30	78.6
24	330	0.20	79.6
25	295	0.25	47.9
26	310	0.40	58.0
27	330	0.50	75.5
28	340	0.25	84.1
29	340	0.30	82.0
30	345	0.10	77.9
31	350	0.15	80.0
32	350	0.50	84.7
33	350	0.20	80.4
34	345	0.50	80.4
35	335	0.30	78.9
36	335	0.15	81.3
37	320	0.15	77.7
38	265	0.45	0.0

a) Conditions: 250–350 °C and 0.1–0.5 mL/min (0.5 M of **1** in MeOH), 100 bar, 0.5 mL/min CO_2. The optimal conditions were verified, and a optimal yield of 86% was found.

Table S3. The optimisation for product 7 starting from 5.[a]

Measurement no	Temperature (°C)	Flow Rate (mL/min)	GC-yield of **7** (%)
1	300	0.25	3.02
2	311	0.28	4.52
3	321	0.49	4.24
4	340	0.18	34.34
5	360	0.27	39.93
6	380	0.40	50.32
7	370	0.10	38.95
8	365	0.50	34.95
9	355	0.50	25.05
10	350	0.50	22.58
11	335	0.50	13.52
12	330	0.10	39.83
13	345	0.10	52.86
14	370	0.15	39.48
15	370	0.25	61.68
16	380	0.10	41.46
17	380	0.25	59.94

a) Conditions: 300–380 °C and 0.1–0.5 mL/min (0.5 M of **5** in MeOH), 100 bar, 0.5 mL/min CO_2. The optimal conditions were verified, and slightly lower yields were obtained: 50–55%.

Table S4. The conditions for the optimisation for product 9 starting from 8.[a]

Measurement no	Temperature (°C)	Flow Rate (mL/min)	GC-yield of **9** (%)
1	300	0.16	0.6
2	317	0.77	1.2
3	332	0.43	2.6
4	349	0.93	9.0
5	365	0.87	6.7
6	380	0.71	11.0
7	360	1.00	6.4
8	355	0.10	13.4
9	340	0.95	7.6
10	325	1.00	10.7
11	310	0.10	3.1
12	305	1.00	6.6
13	330	1.00	7.1
14	350	0.80	7.0
15	360	0.50	3.6
16	370	0.10	15.3
17	380	0.10	8.7
18	380	0.50	10.8
19	375	1.00	2.8
20	375	0.85	4.3
21	375	0.45	11.0
22	360	0.10	12.5
23	345	0.35	2.5

a) Conditions: 300–380 °C and 0.1–1.0 mL/min (1 M of **8** in MeOH), 100 bar, 0.5 mL/min CO_2. This optimisation was troublesome as very low yields were seen throughout, and also at some instances false responses from the analysis. The 'optimal conditions' were verified to be 370 °C, 0.1 mL/min giving a 11% yield.

Table S5. The conditions for the optimisation for product 10 starting from 8.[a]

Measurement no	Temperature (°C)	Flow Rate (mL/min)	GC-yield of **10** (%)
1	300	0.16	0.0
2	317	0.77	0.0
3	333	0.43	10.5
4	349	0.93	5.0
5	365	0.87	12.7
6	380	0.71	33.4
7	360	1.00	6.3
8	355	0.10	38.2
9	340	0.95	5.5
10	325	1.00	4.2
11	310	0.10	26.0
12	305	1.00	7.5
13	330	0.10	26.2
14	345	0.20	19.9
15	360	0.50	16.1
16	370	0.10	30.0
17	380	0.10	18.0
18	380	0.40	34.9
19	380	0.65	22.6
20	380	0.85	18.9
21	375	0.60	21.5
22	370	0.35	42.6
23	365	0.15	48.3
24	350	0.10	31.8
25	345	0.35	11.2
26	325	0.10	19.5
27	340	0.10	26.9
28	350	0.45	11.0
29	360	0.10	57.1
30	360	0.25	24.2
31	370	0.25	40.0
32	375	0.35	34.4
33	380	0.25	50.7
34	370	1.00	10.4
35	365	0.50	19.1
36	365	0.40	23.0
37	360	0.30	26.6
38	360	0.15	51.9
39	325	0.35	9.6
40	315	0.35	0.0
41	335	0.10	25.7
42	355	0.15	44.8
43	365	0.10	70.8
44	365	0.30	24.3
45	380	0.30	35.9
46	380	0.80	19.8
47	380	0.50	34.7
48	375	0.20	57.6
49	370	0.50	19.0
50	360	0.45	16.7
51	345	0.10	38.2
52	315	0.10	20.2
53	305	0.80	9.0

54	305	0.10	36.0
55	305	0.70	11.4
56	350	0.20	24.9
57	350	0.30	18.1
58	360	0.75	10.2
59	375	0.45	33.1
60	380	0.15	50.3
61	380	0.25	43.3

a) Conditions: 300–380 °C and 0.1–1.0 mL/min (1 M of **8** in MeOH), 100 bar, 0.5 mL/min CO_2. The optimal conditions were verified, and slightly lower yields were obtained: 57–63%.

Table S6. The conditions for the optimisation for 9 starting from 12.[a]

Measurement no	Temperature (°C)	Flow Rate (mL/min)	GC-yield of **9** (%)
1	300	0.16	14.0
2	317	0.77	10.9
3	333	0.43	18.7
4	348	0.93	19.3
5	365	0.87	31.1
6	379	0.71	41.5
7	360	1.00	25.7
8	355	0.10	43.1
9	340	0.95	16.7
10	325	1.00	13.8
11	310	0.10	13.7
12	305	1.00	13.7
13	345	0.20	24.8
14	360	0.50	31.7
15	370	0.10	51.5
16	380	0.10	68.2
17	380	0.40	57.3
18	380	0.50	44.7
19	380	0.80	38.8
20	380	0.30	63.3
21	380	0.15	59.0
22	375	0.40	51.7
23	365	0.10	58.6
24	360	0.65	28.2
25	345	0.35	23.4
26	335	0.15	29.7
27	360	0.10	30.6
28	365	0.20	36.8
29	375	0.10	53.6
30	375	0.45	37.9
31	380	0.25	46.6
32	375	0.45	36.5
33	376	0.30	46.0
34	374	0.15	48.3
35	360	0.35	34.9
36	350	0.45	29.7
37	350	0.10	42.4
38	325	0.35	20.9
39	365	0.30	42.2
40	370	0.15	55.1

41	375	0.45	42.4
42	380	0.35	50.8
43	380	0.55	45.1
44	380	0.45	51.0
45	375	0.65	37.9
46	375	0.25	39.5
47	370	0.35	44.9
48	360	0.75	27.1
49	330	0.10	48.9
50	325	0.10	36.3
51	345	0.50	23.8
52	370	0.45	40.6
53	375	0.20	53.6
54	375	0.35	51.9
55	380	0.60	43.4
56	380	0.20	53.2
57	375	0.55	39.1
58	370	0.70	39.5
59	370	0.25	55.6
60	365	0.40	41.0
61	335	0.10	49.0
62	325	0.45	21.7
63	340	0.10	38.5
64	355	0.75	0.0

a) Conditions: 300–380 °C and 0.1–1.0 mL/min (0.5 M of **12** in MeOH), 100 bar, 0.5 mL/min CO_2. During the verification experiment, 46% of **9** was found at 0.3 mL/min, 380 °C.

Table S7. The conditions for the optimisation for 10 starting from 15.[a]

Measurement no	Temperature (°C)	Flow Rate (mL/min)	GC-yield of **10** (%)
1	300	0.16	59.6
2	317	0.77	30.3
3	333	0.43	47.0
4	349	0.93	58.9
5	365	0.87	64.3
6	379	0.71	72.8
7	360	1.00	64.8
8	355	0.10	52.8
9	340	0.95	49.6
10	325	1.00	27.7
11	310	0.10	63.5
12	305	1.00	24.1
13	330	0.10	54.1
14	355	0.90	62.8
15	360	0.50	67.1
16	371	0.10	30.8
17	375	0.50	68.4
18	380	0.10	32.9
19	380	1.00	67.9
20	380	0.65	70.5
21	375	0.90	63.8
22	370	1.00	63.2
23	370	0.45	64.2
24	365	0.55	64.9

25	350	0.30	66.1
26	345	0.35	58.9
27	325	0.35	50.0
28	355	0.40	64.0
29	370	0.55	64.0
30	375	0.65	64.8
31	380	0.50	64.7
32	380	0.80	62.8
33	375	0.55	65.6
34	375	0.40	69.5
35	355	1.00	58.9
36	355	0.50	62.2
37	355	0.25	67.3
38	340	0.45	63.8
39	320	0.10	49.3
40	350	0.55	59.0
41	359	0.25	65.8
42	361	0.65	64.6
43	360	0.75	64.5
44	365	0.45	68.3
45	365	0.80	66.7
46	380	0.55	72.0
47	380	0.15	46.2

a) Conditions: 300–380 °C and 0.1–1.0 mL/min (0.5 M of **15** in MeOH), 100 bar, 0.5 mL/min CO_2. During the verification experiment 68% yields were found at 380 °C, 1 mL/min.

2. NMR characterisation data

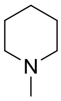

2b ^{1}H NMR ($CDCl_3$, 400 MHz) δ = 2.34 – 2.31 (m, 7H), 1.62 – 1.56 (m, 4H), 1.53 – 1.33 (m, 2H). ^{13}C NMR ($CDCl_3$, 100 MHz) δ = 56.5, 46.7, 25.8, 23.7.

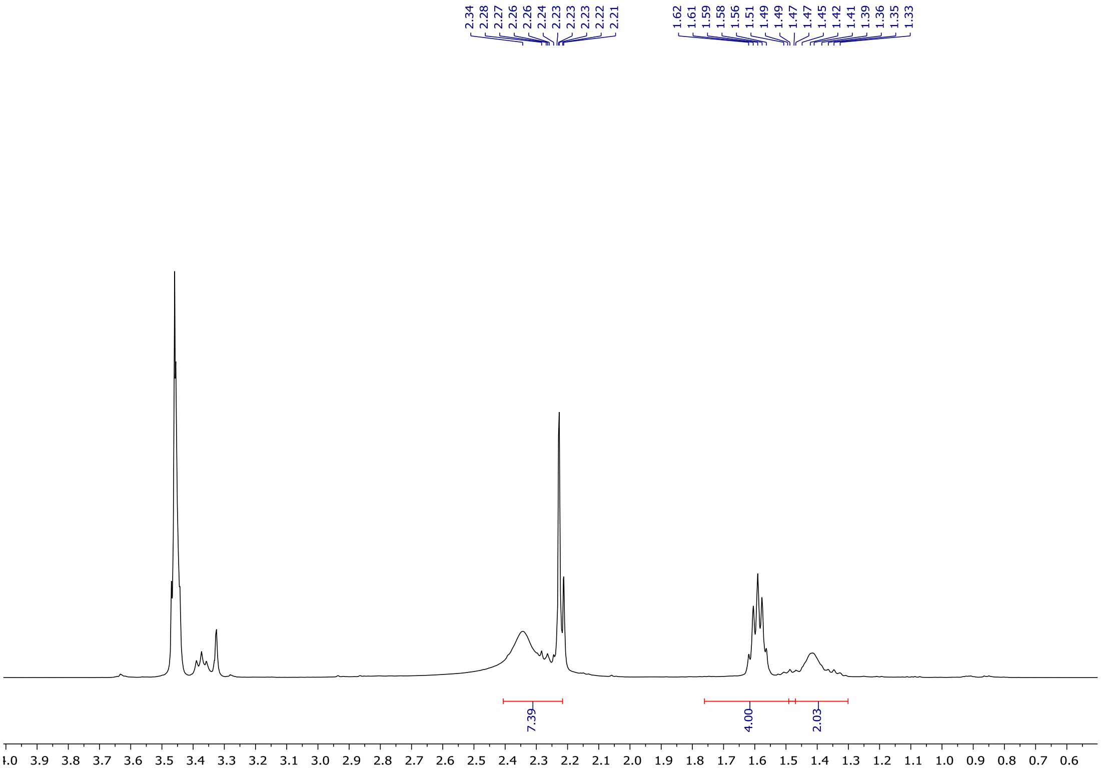

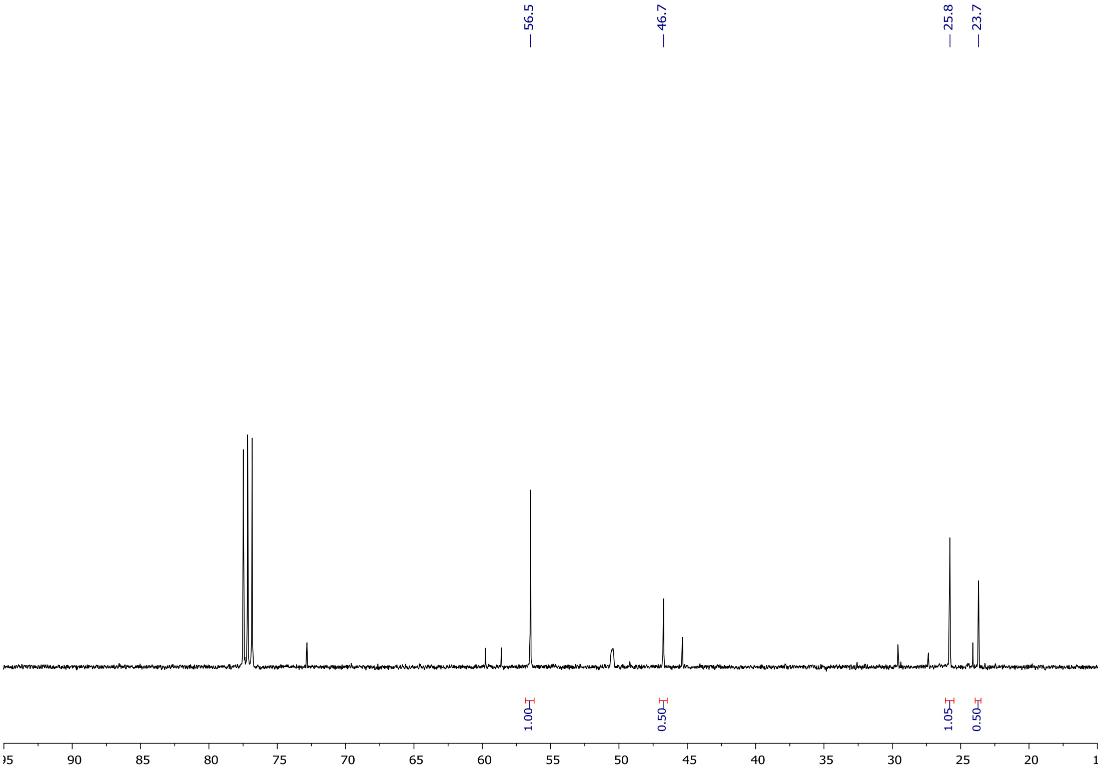

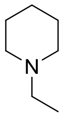

2c ^{1}H NMR (MeOD, 300 MHz) δ = 3.49 (q, *J* = 7.0 Hz, 2H), 2.51 – 2.43 (m, 4H), 1.68 – 1.60 (m, 4H), 1.52 – 1.47 (m, 2H), 1.11 (t, *J* = 7.0 Hz, 3 H). ^{13}C NMR (MeOD, 75 MHz) δ =66.9, 54.8, 53.8, 26.2, 25.0, 15.5, 11.6.

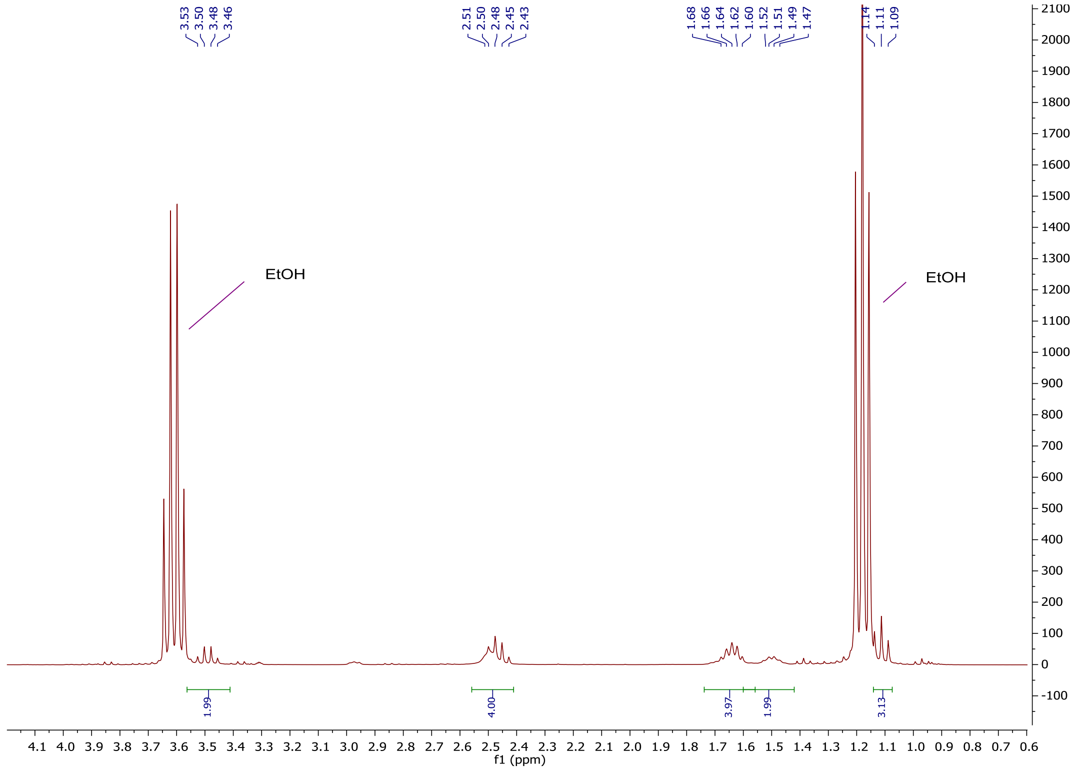

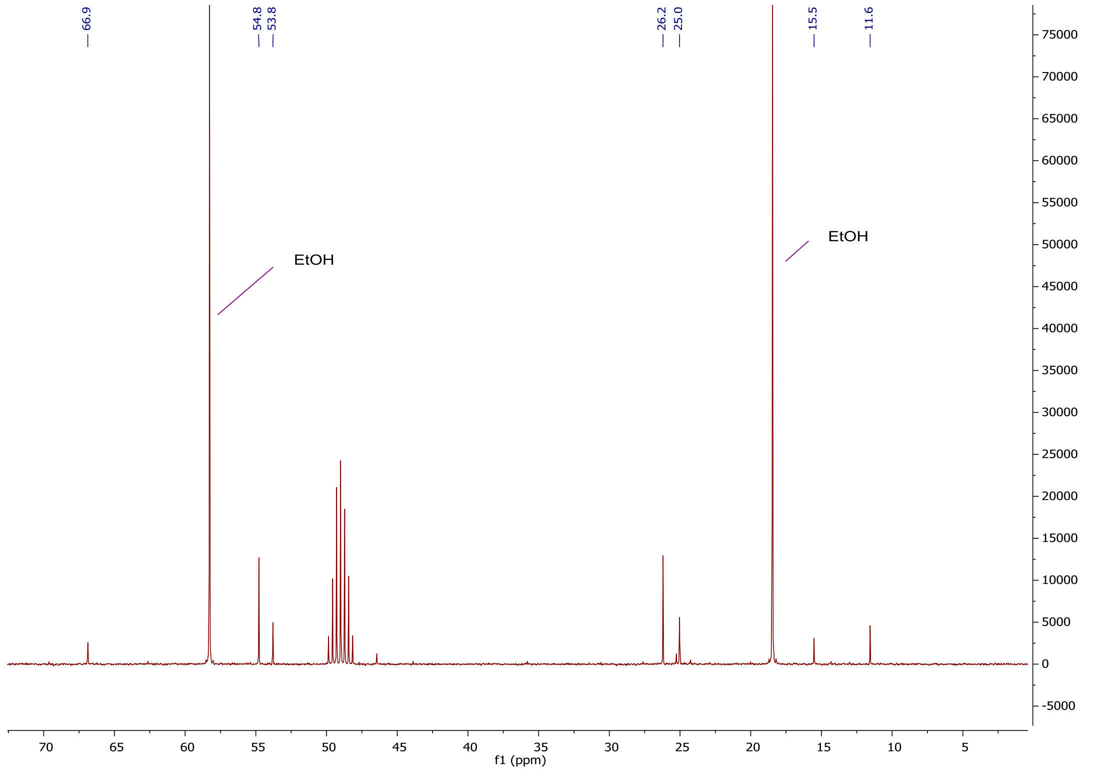

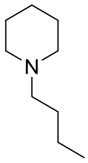

2d ^{1}H NMR ($CDCl_3$, 300 MHz) δ = 2.63 – 2.06 (m, 16H), 0.98 – 0.93 (m, 3H). ^{13}C NMR ($CDCl_3$, 75 MHz) δ = 59.1, 54.2, 28.3, 25.9, 25.2, 24.1, 20.7.

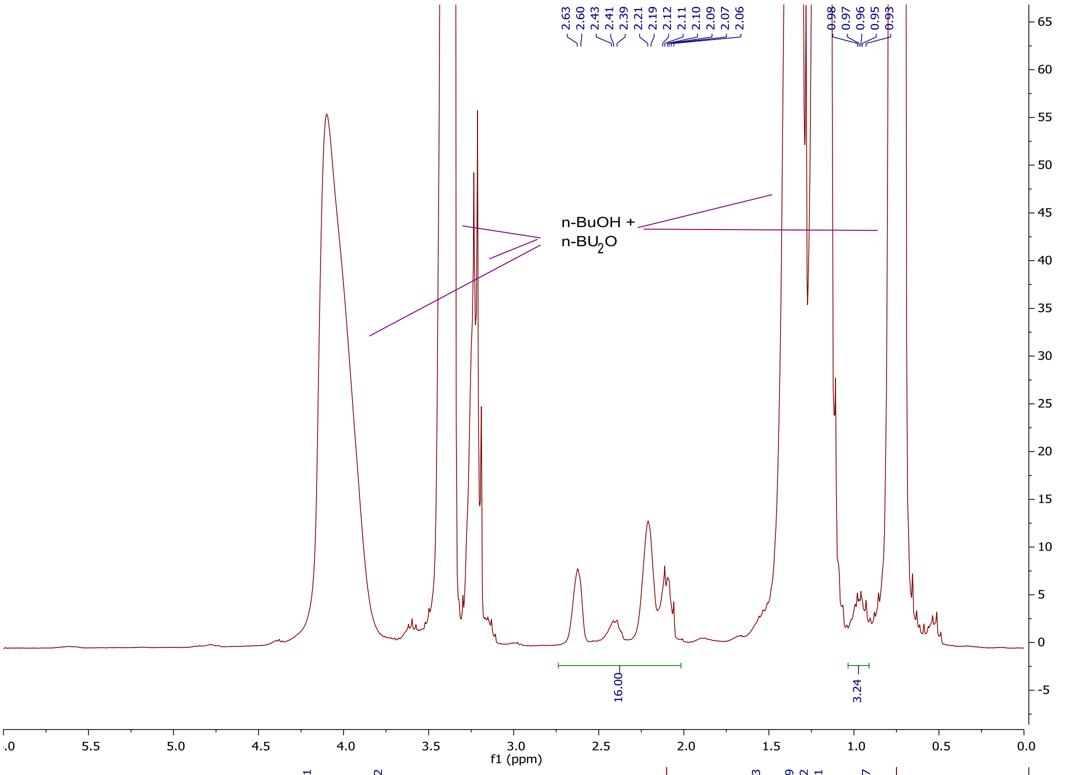

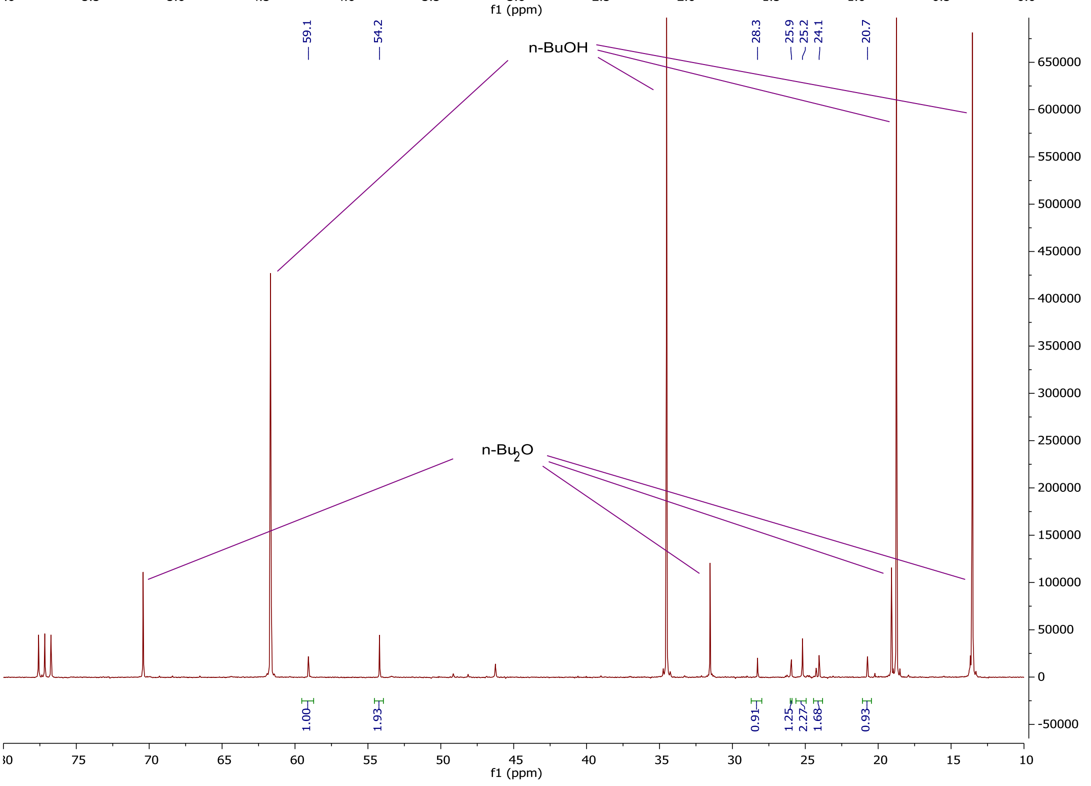

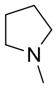

4 ^{1}H NMR ($CDCl_3$, 400 MHz) δ = 2.55 – 2.53 (m, 4H), 2.34 (s, 3H), 1.77 – 1.75 (m, 4H). ^{13}C NMR ($CDCl_3$, 100 MHz) δ = 55.7, 41.5, 23.7.

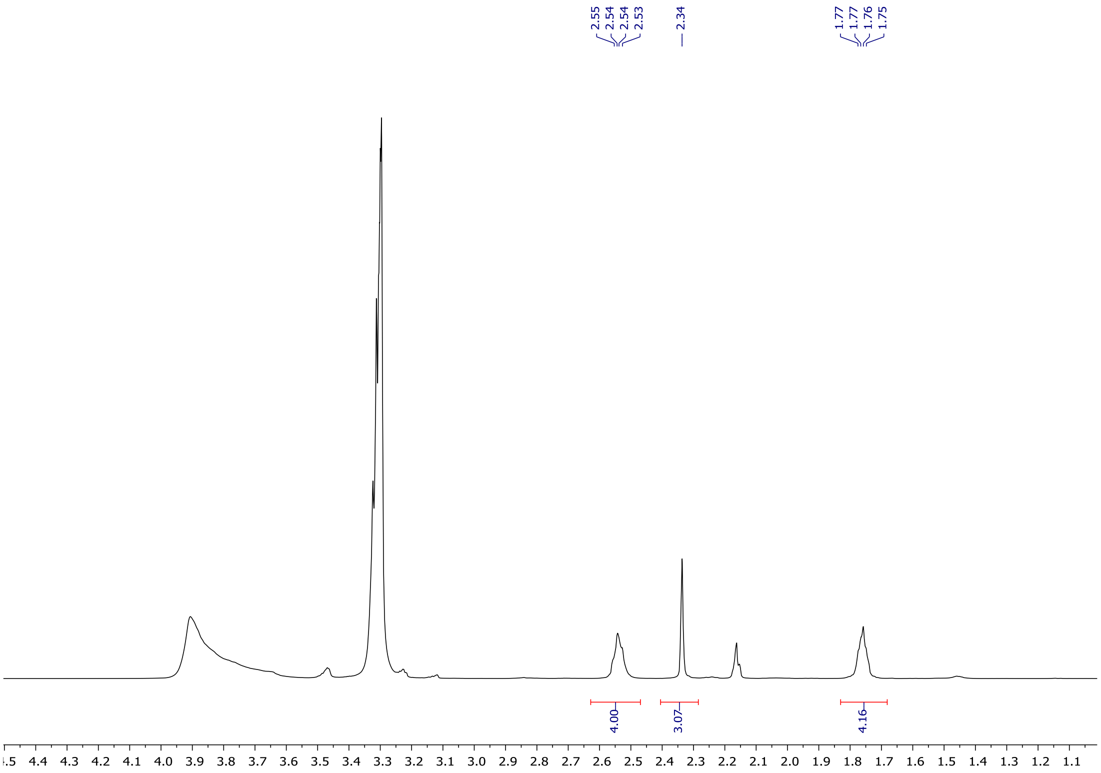

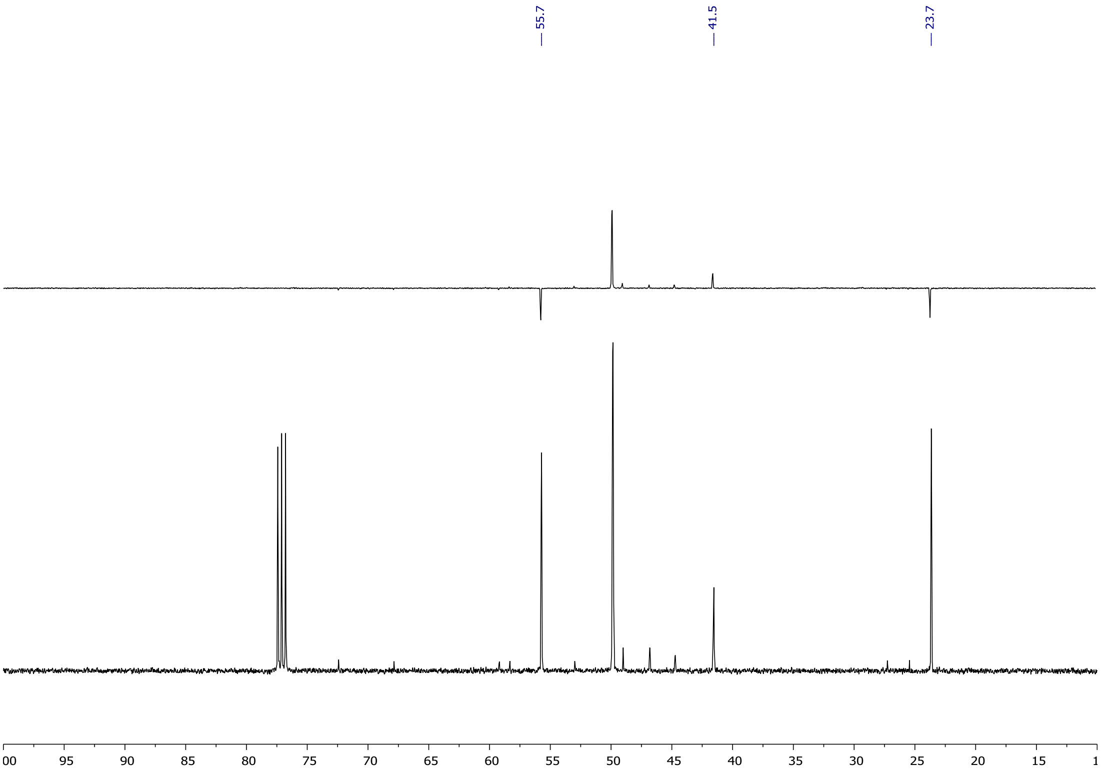

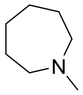

6 ^{1}H NMR ($CDCl_3$, 400 MHz) δ = 2.62 – 2.59 (m, 4H), 2.34 (s, 3H), 1.68 – 1.65 (m, 8H). ^{13}C NMR ($CDCl_3$, 100 MHz) δ = 58.5, 46.5, 27.3, 26.6.

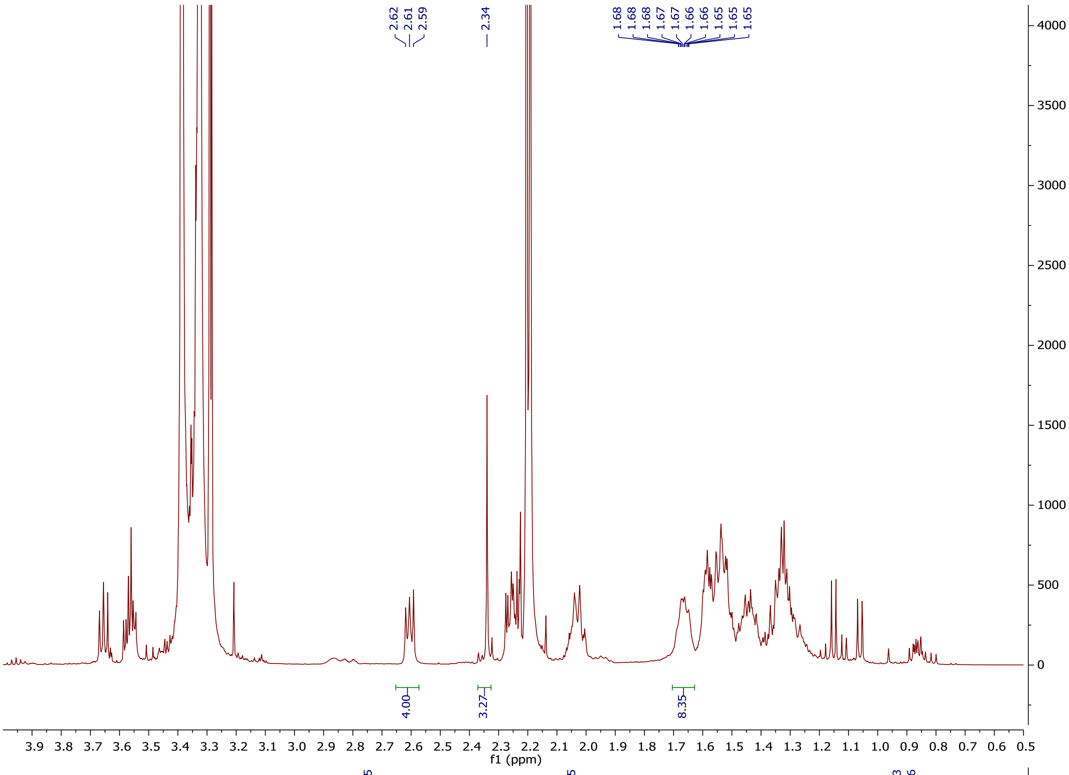

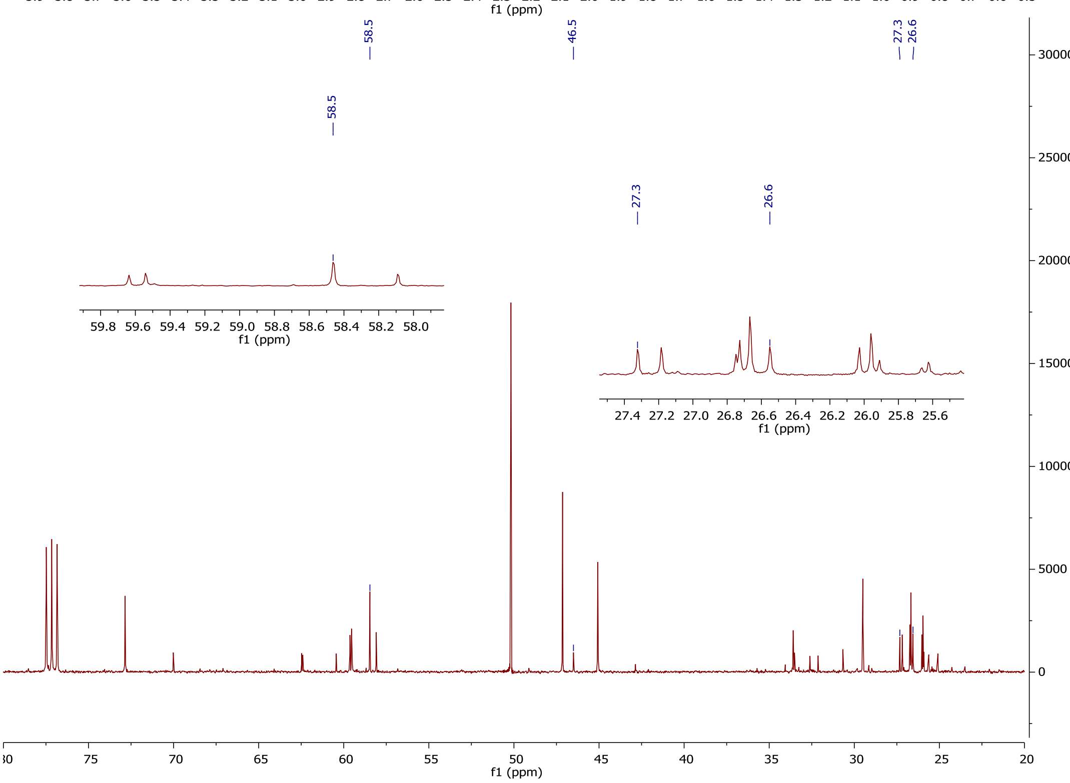

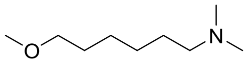

7 ^{1}H NMR (MeOD$_3$, 400 MHz) δ = 3.44 (t, *J* = 8.0 Hz, 2H), 3.36 (s, 3H), 2.38 – 2.34 (m, 2H), 2.27 (s, 6H), 1.63 – 1.50 (m, 4H), 1.43 – 1.34 (m, 4H). ^{13}C NMR (MeOD, 100 MHz) δ = 73.7, 60.4, 58.7, 45.2, 30.2, 28.2, 27.9, 26.8.

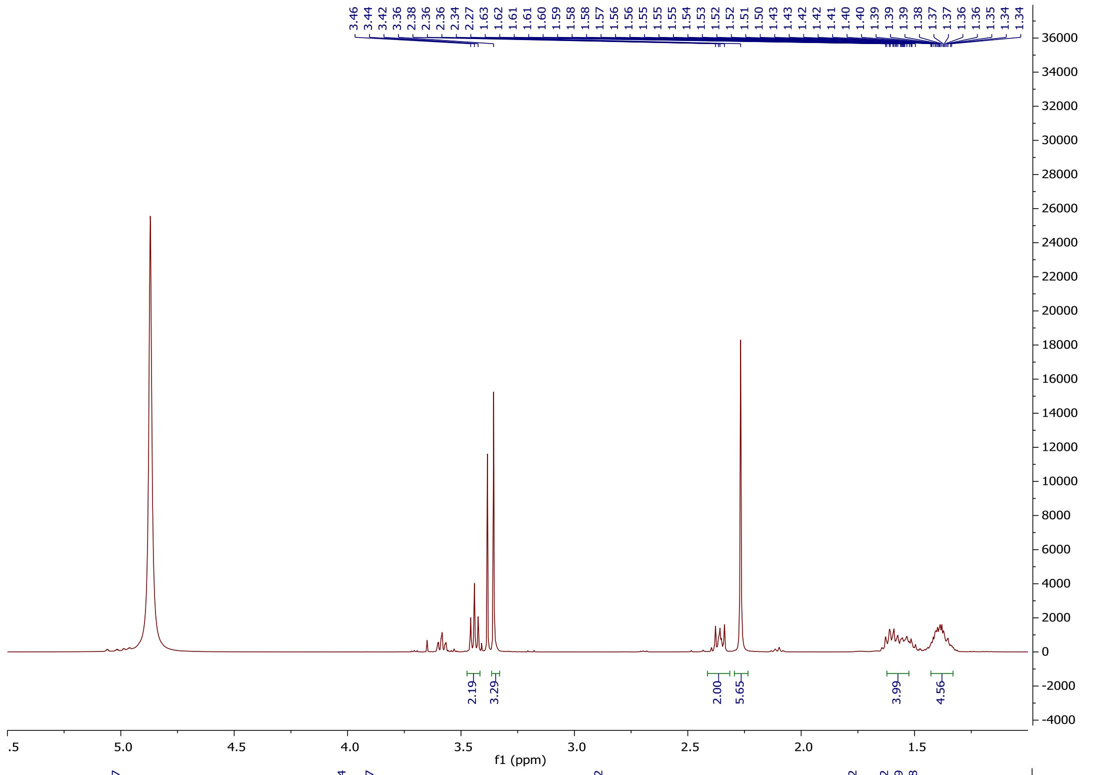

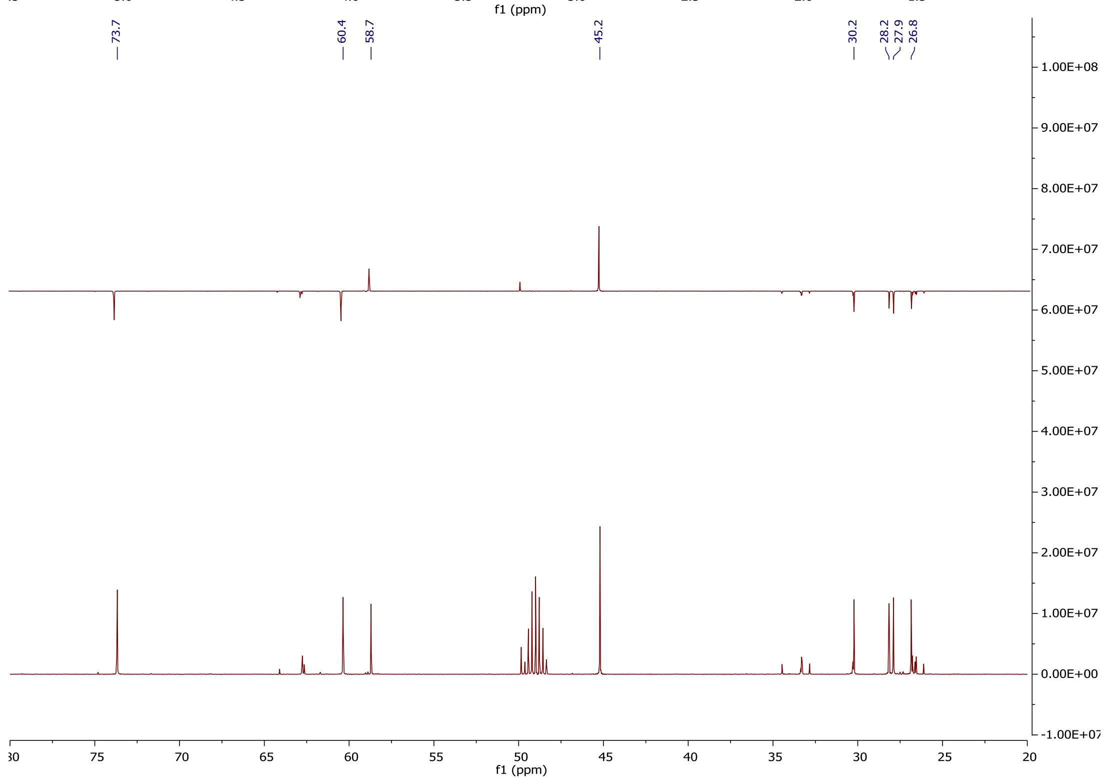

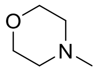

9 ^{1}H NMR ($CDCl_3$, 300 MHz) δ = 3.74 – 3.71 (m, 4H), 2.43 – 2.40 (m, 4 H), 2.29 (s, 3H). ^{13}C NMR ($CDCl_3$, 75 MHz) δ = 66.9, 55.4, 46.4.

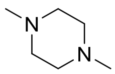

10 ^{1}H NMR ($CDCl_3$, 300 MHz) δ = 2.60 – 2.33 (m, 8 H), 2.29 (s, 6H). ^{13}C NMR ($CDCl_3$, 75 MHz) δ = 55.0, 46.0.

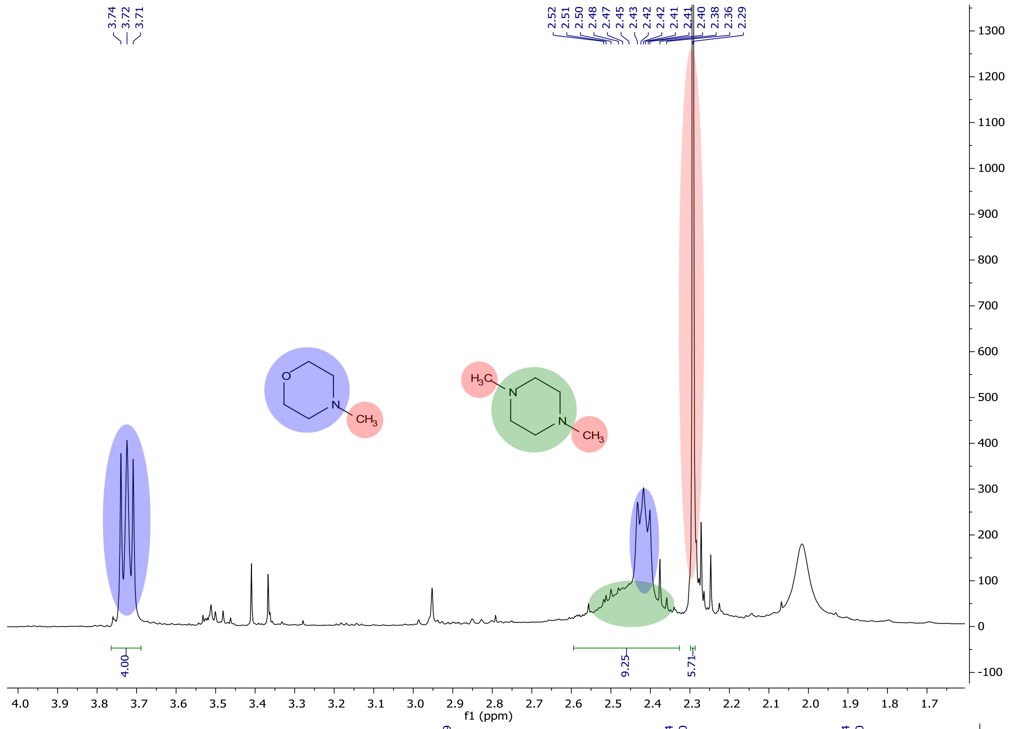

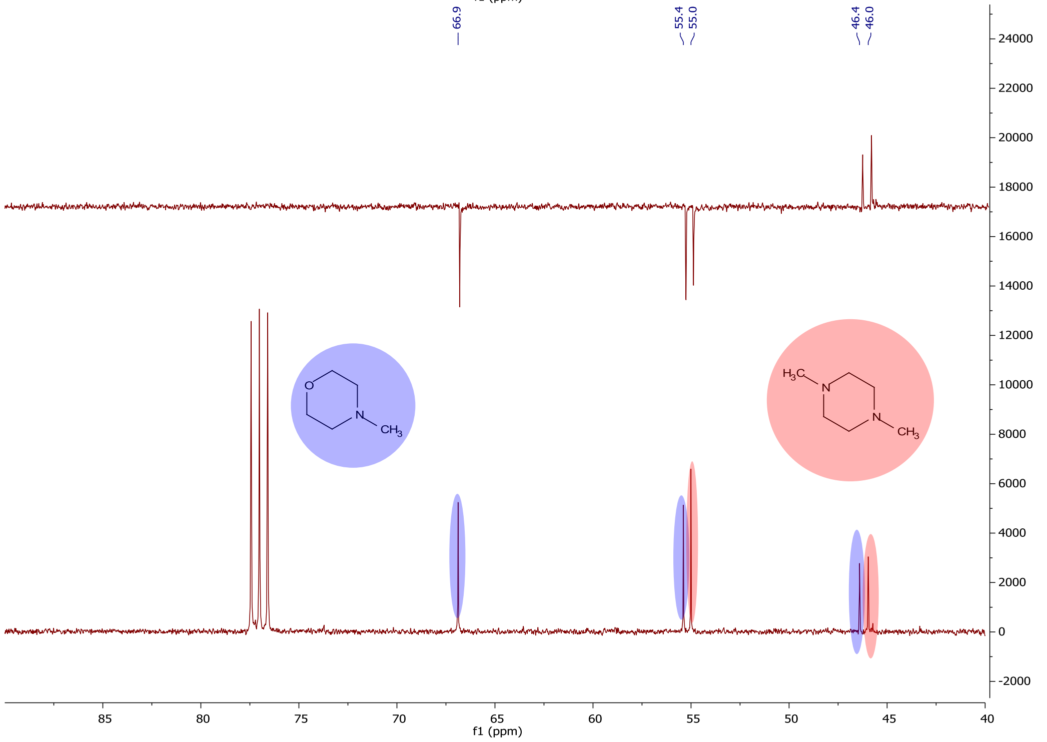

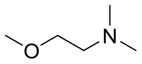

11 ^{1}H NMR ($CDCl_3$, 300 MHz) δ = 3.41 (t, J = 6.0 Hz, 2H), 3.26 (s, 3H), 2.44 (t, J = 6.0 Hz, 2 H), 2.18 (s, 3H). ^{13}C NMR ($CDCl_3$, 75 MHz) δ =70.2, 58.6, 58.3, 45.3.

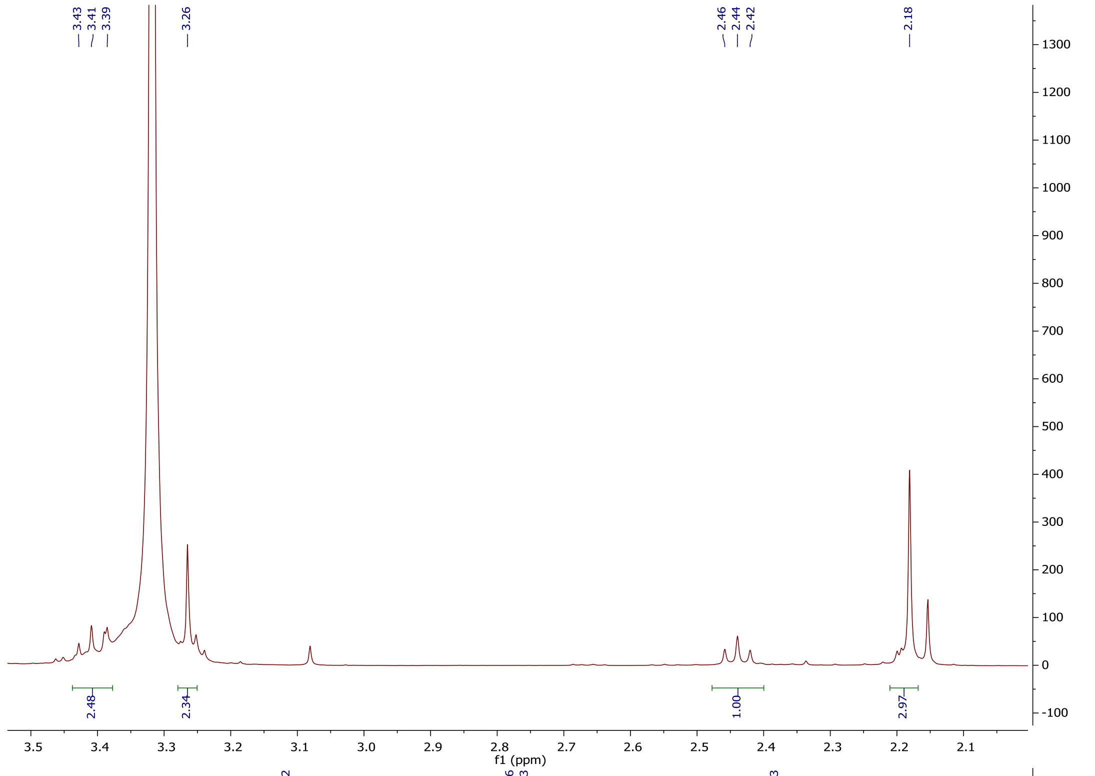

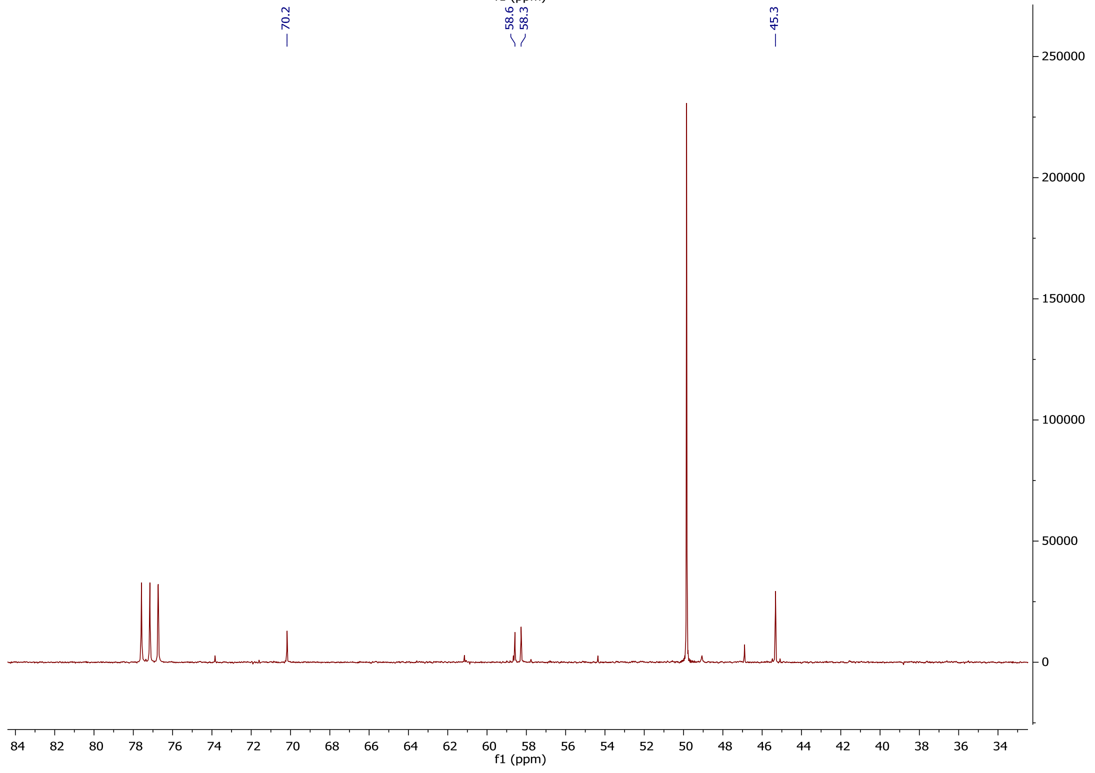

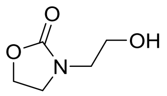

13 ^{1}H NMR ($CDCl_3$, 400 MHz) δ = 4.28 (t, J = 8.0 Hz, 2H), 3.67 (t, J = 4.0 Hz, 2H), 3.65 – 3.62 (m, 2H), 3.31 (t, J = 8.0 Hz, 2H). ^{13}C NMR ($CDCl_3$, 100 MHz) δ = 159.3, 62.2, 59.6, 46.7, 45.5.

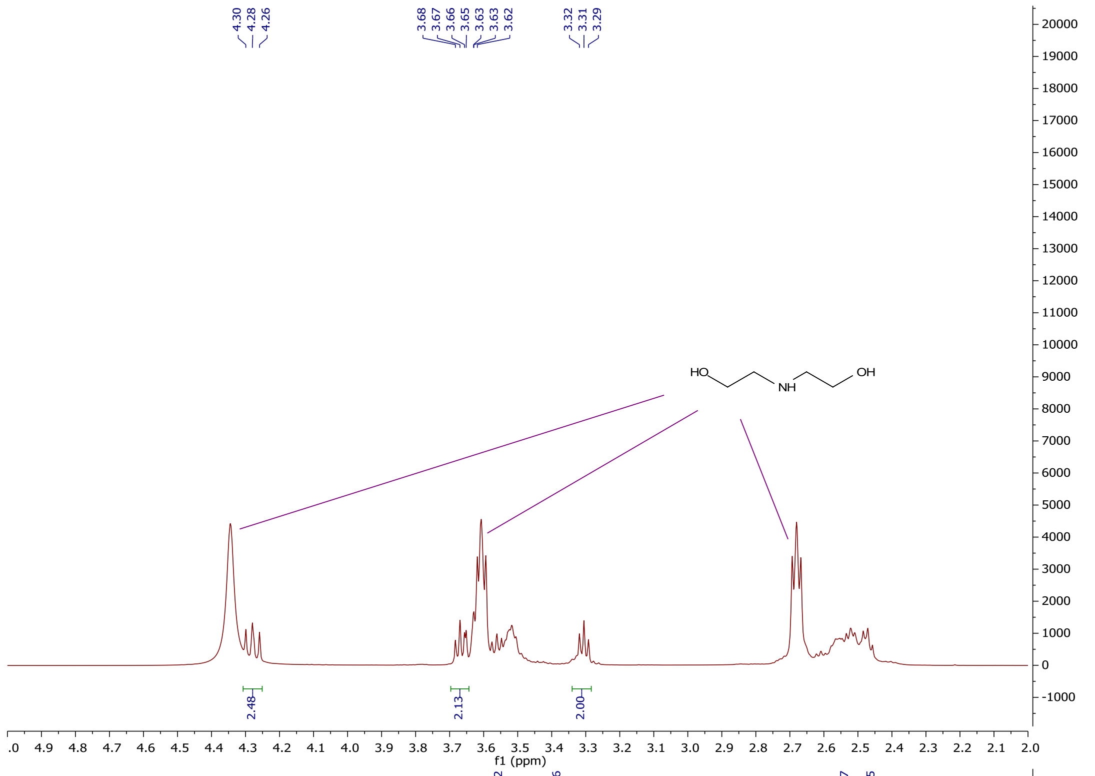

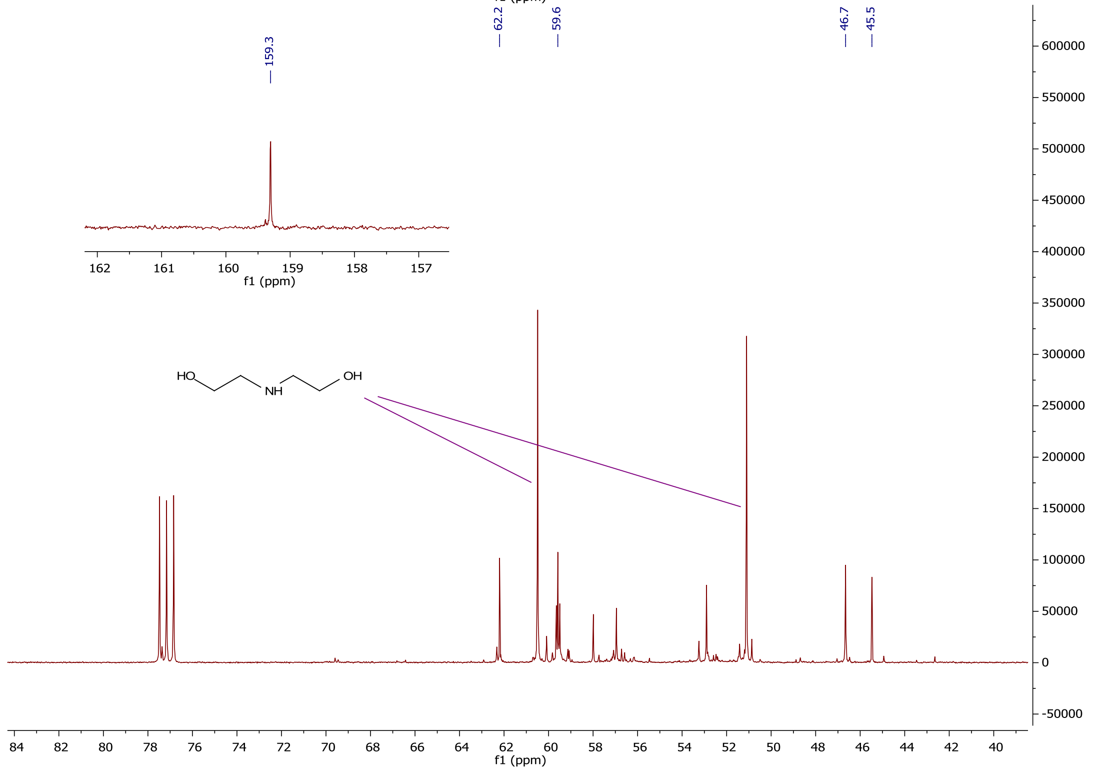

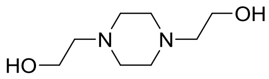

14 ^{1}H NMR ($CDCl_3$, 300 MHz) δ = 3.64 – 3.31 (m, 8H), 2.72 – 2.39 (m, 8H). ^{13}C NMR ($CDCl_3$, 75 MHz) δ = 59.6, 57.9, 53.0.

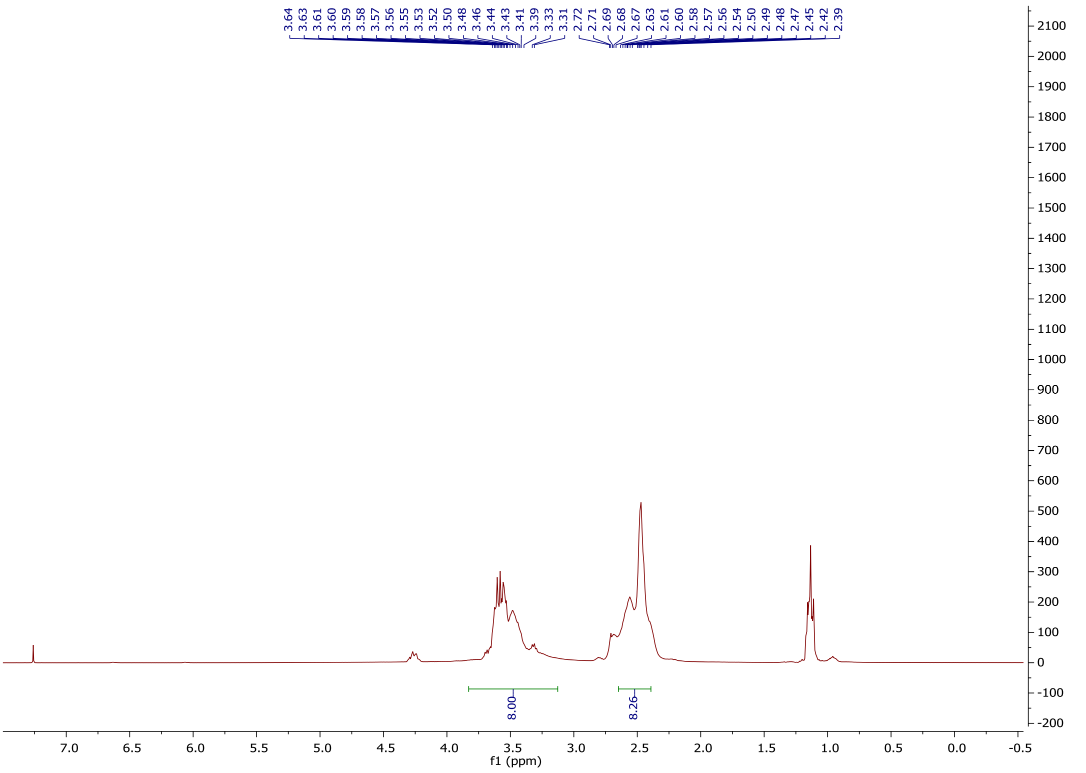

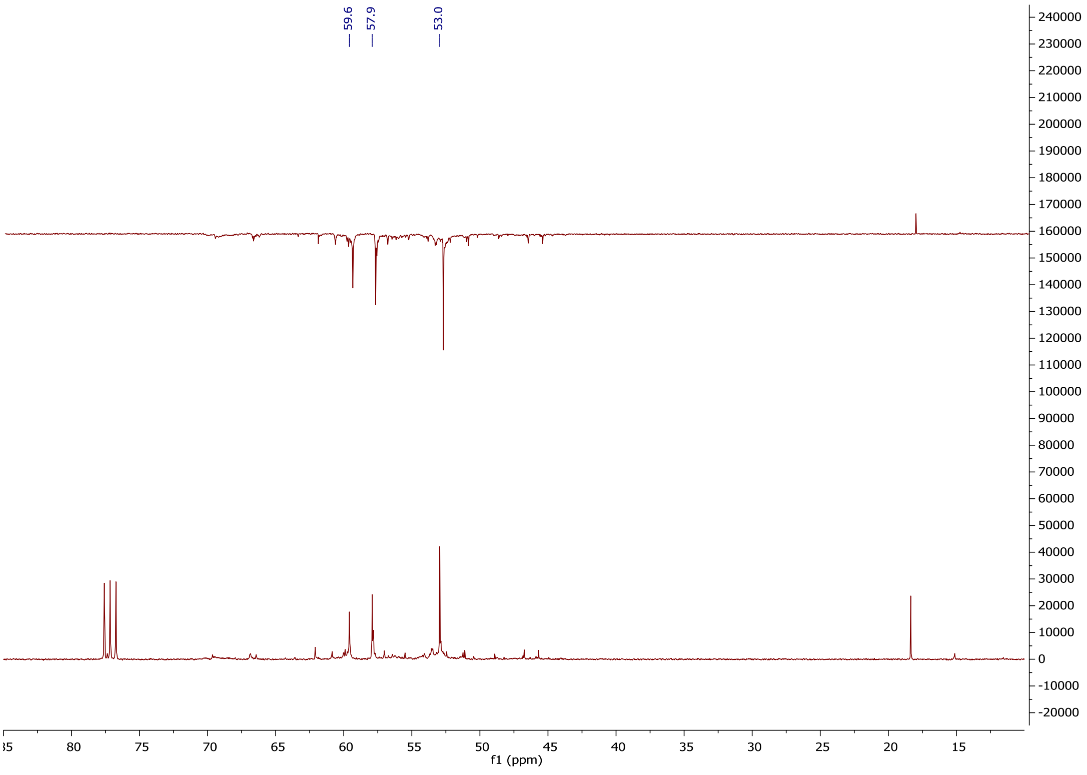

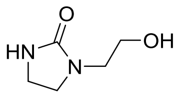

16 ^{1}H NMR ($CDCl_3$, 400 MHz) δ = 5.58 (s, 1H), 3.59 (t, J = 6.0 Hz, 2H), 3.45 – 3.41 (m, 2H), 3.34 – 3.30 (m, 2H), 3.18 (t, J = 6.0 Hz, 2H). ^{13}C NMR ($CDCl_3$, 100 MHz) δ = 163.9, 60.1, 46.3, 45.9, 38.3.

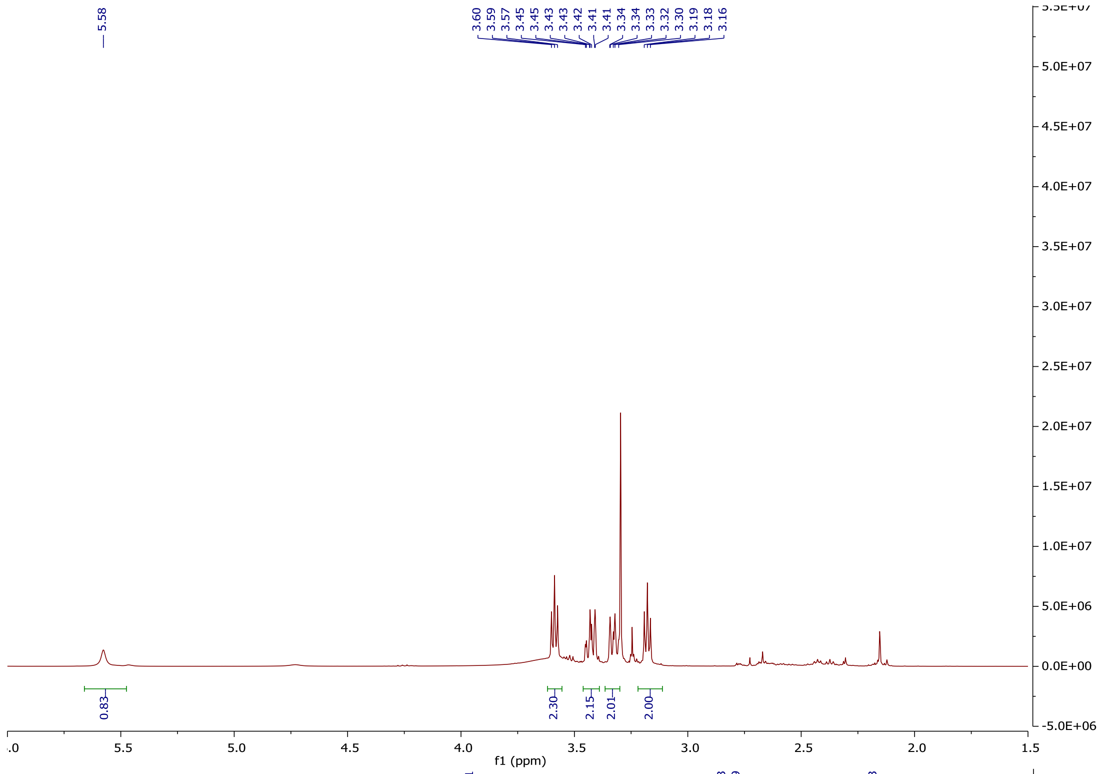

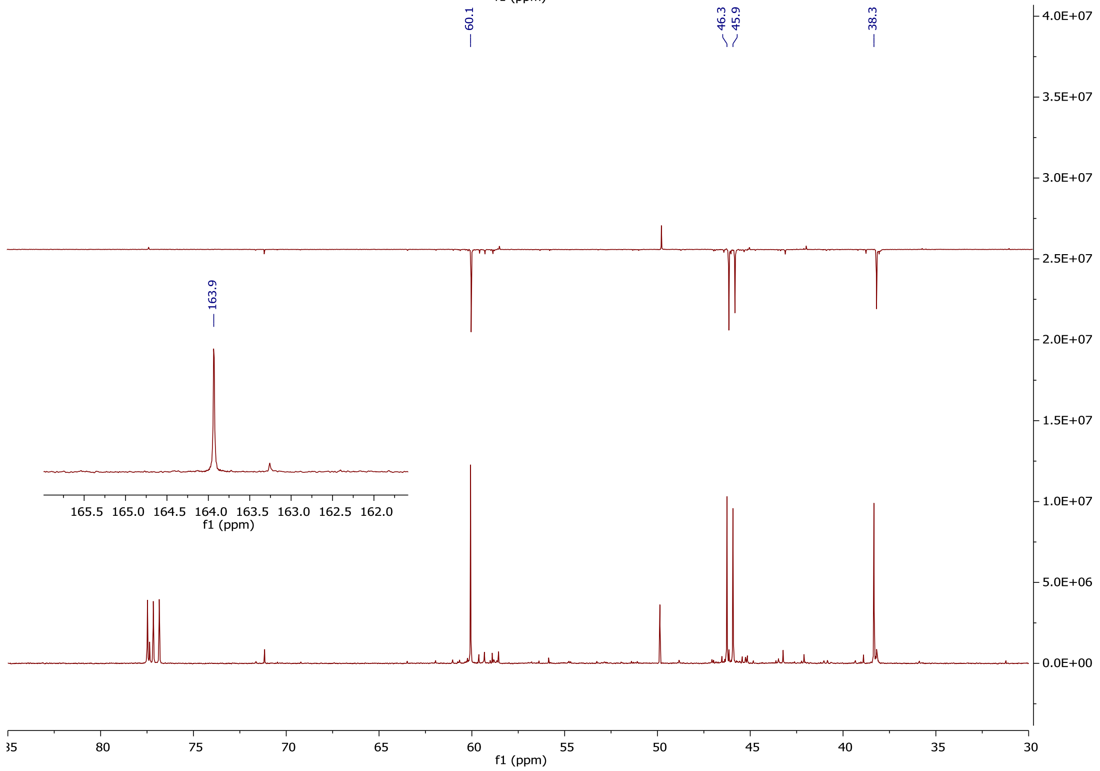

3. GC traces of reaction mixtures

Optimisation of **2a** from **1**.

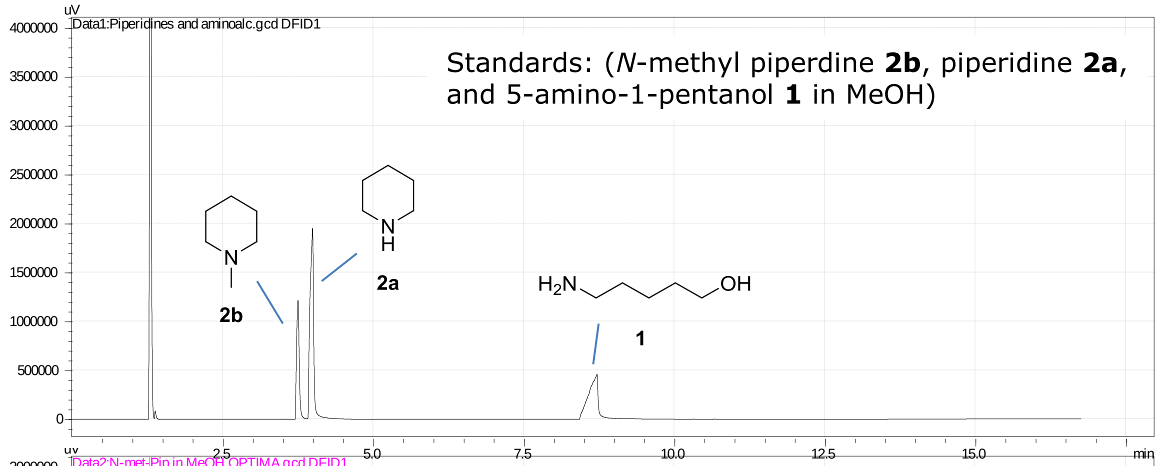

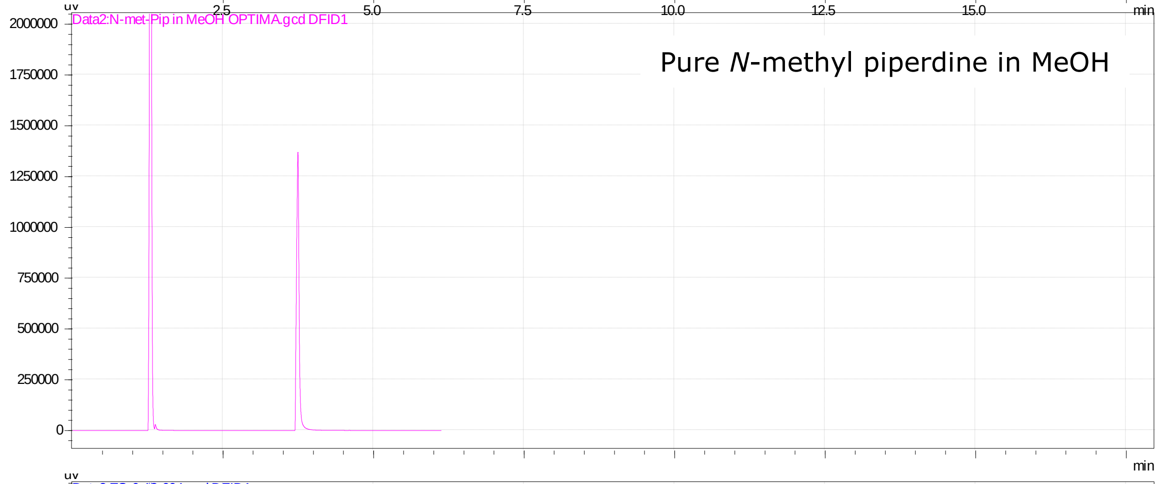

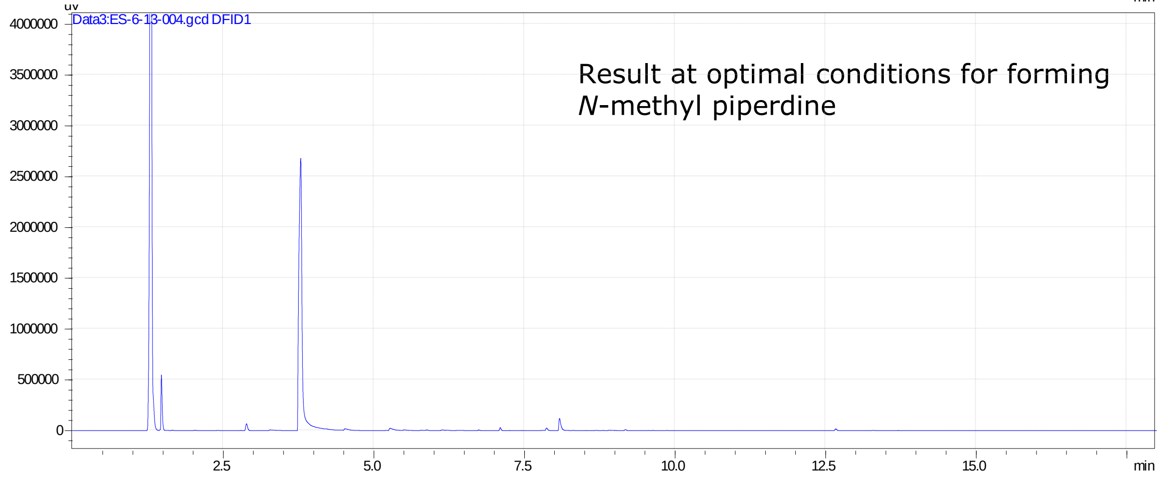

Formation of **2c** from **1**.

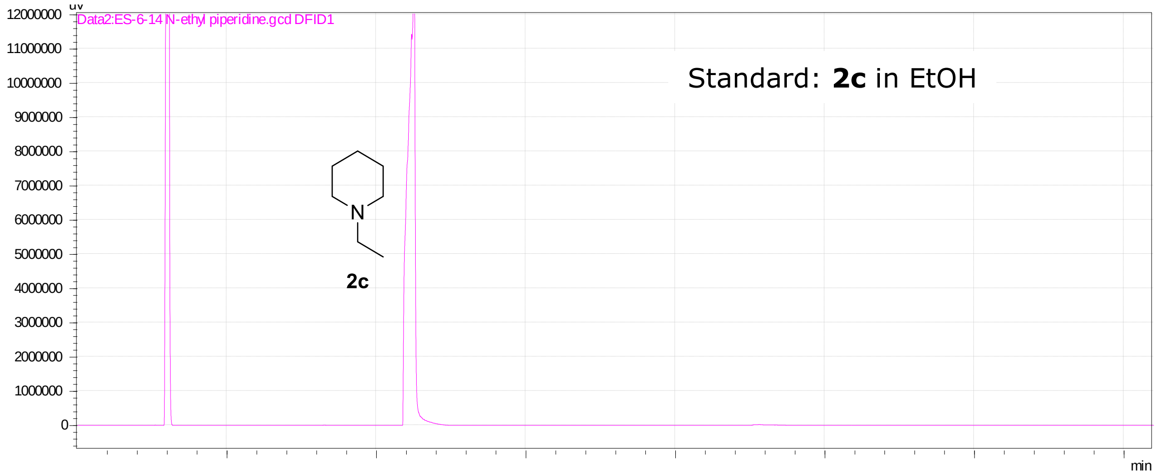

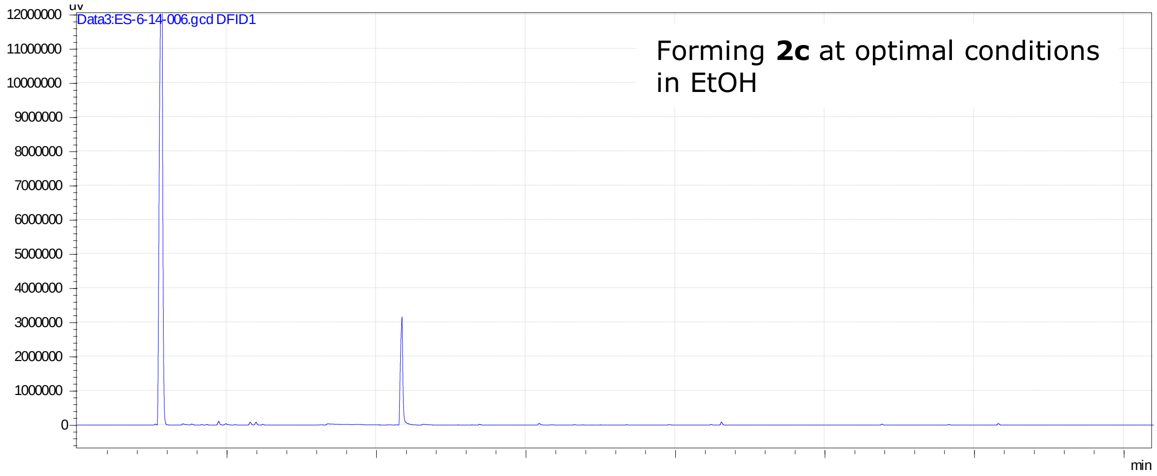

Formation of **2d** and dibutyl ether from **1**.

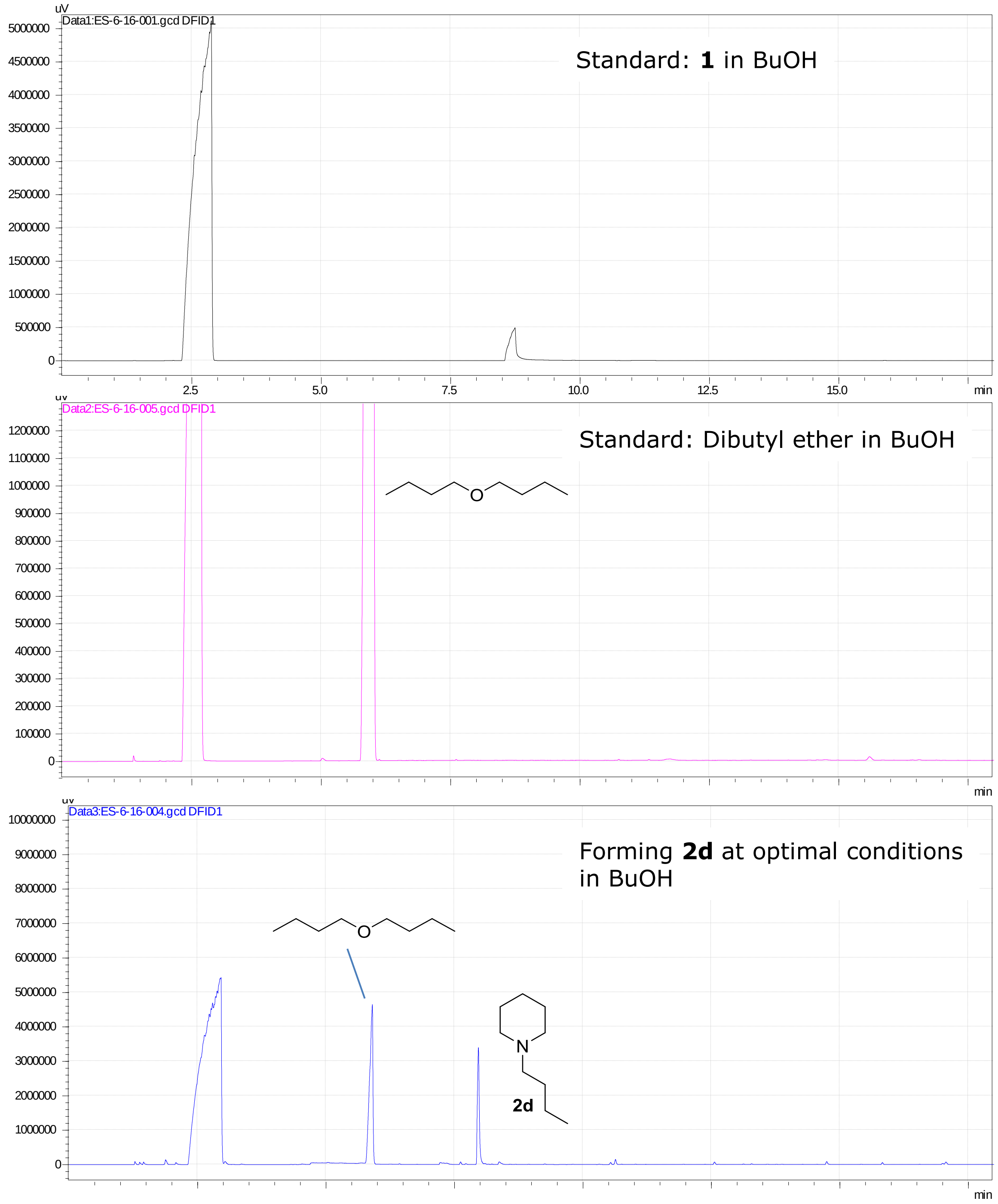

Formation of **4** from **3**.

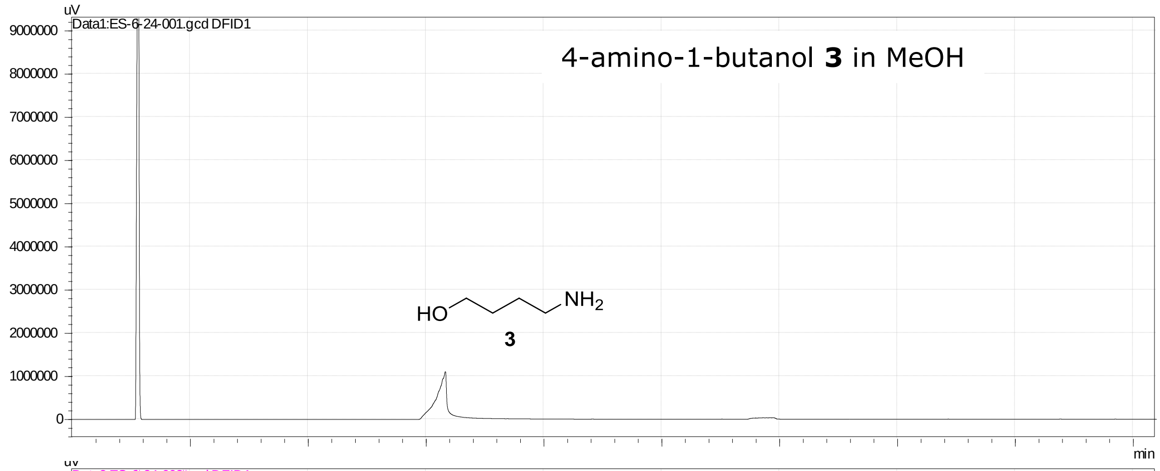

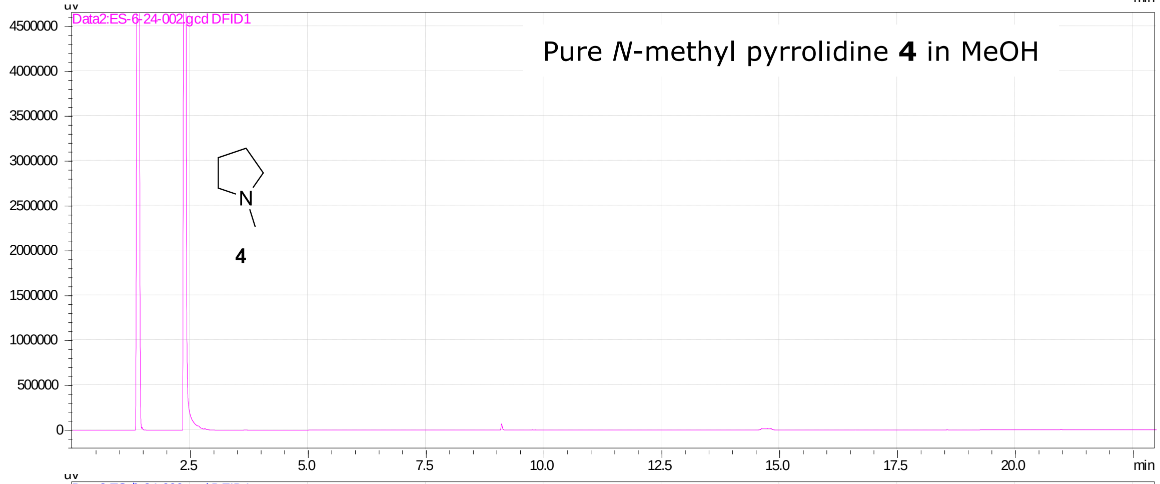

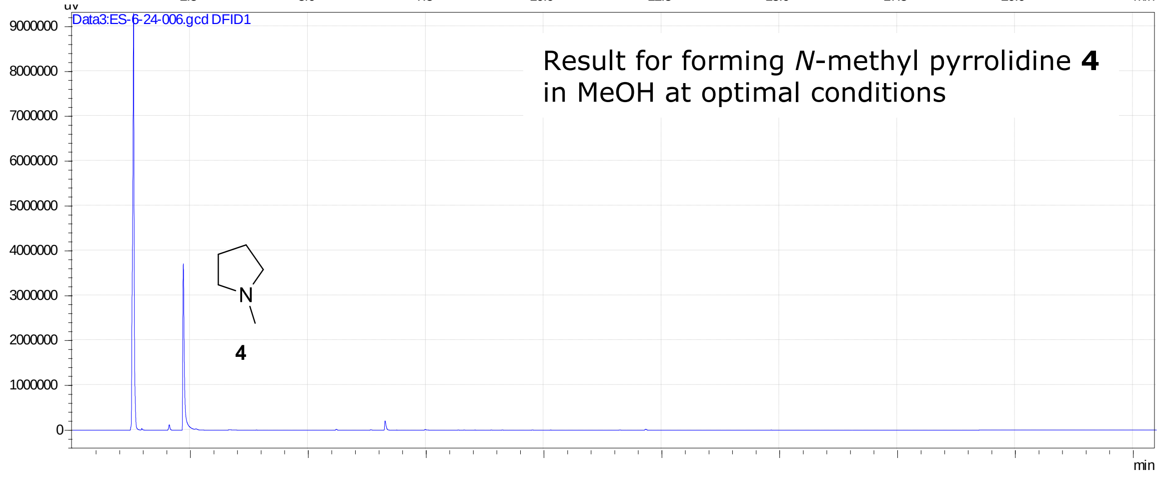

Formation of **6** and **7** from **5**.

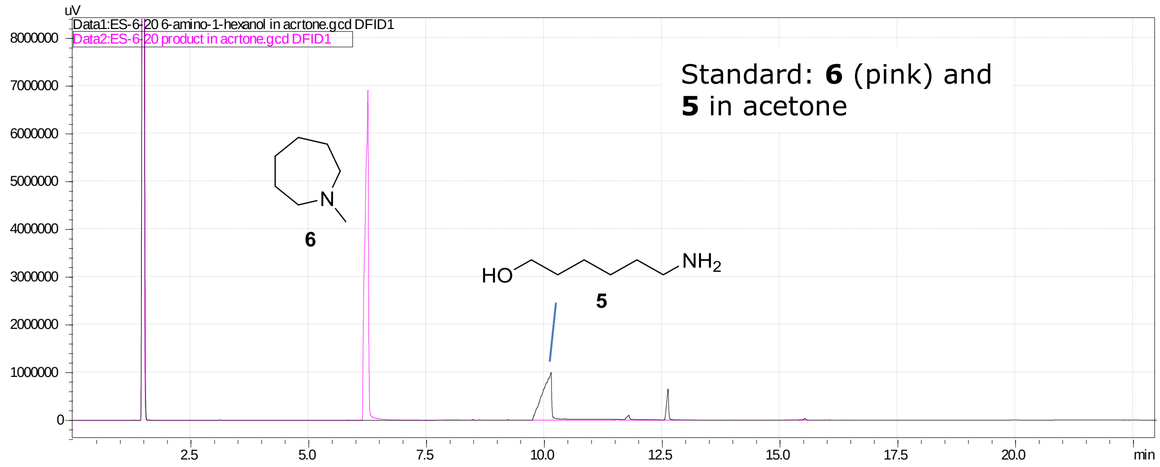

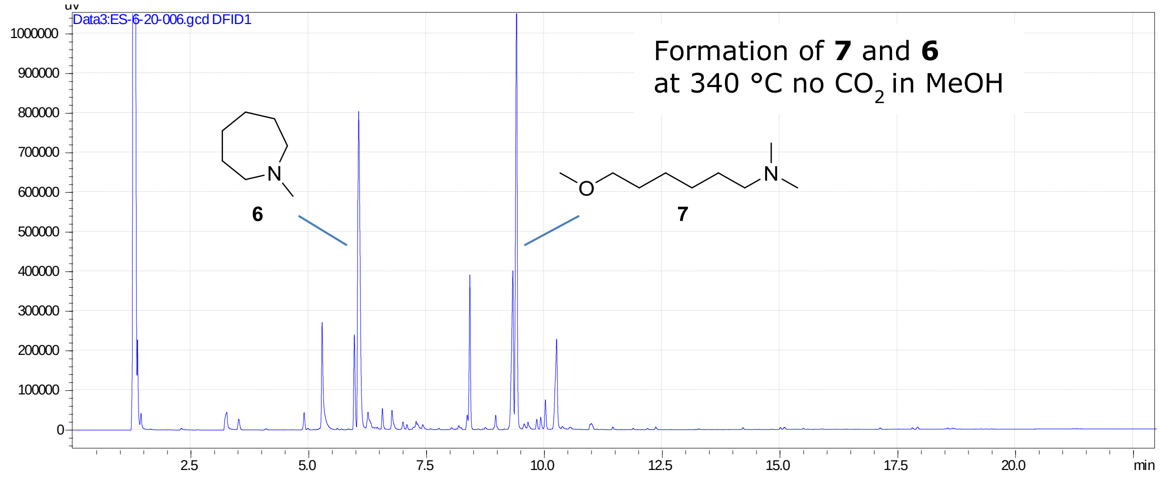

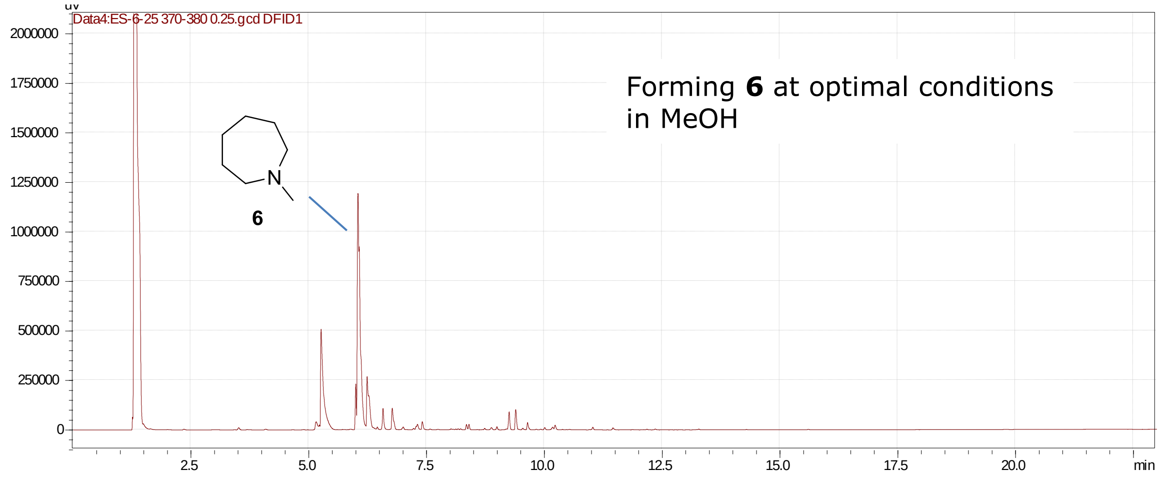

Formation of **10** and **11** from **8**.

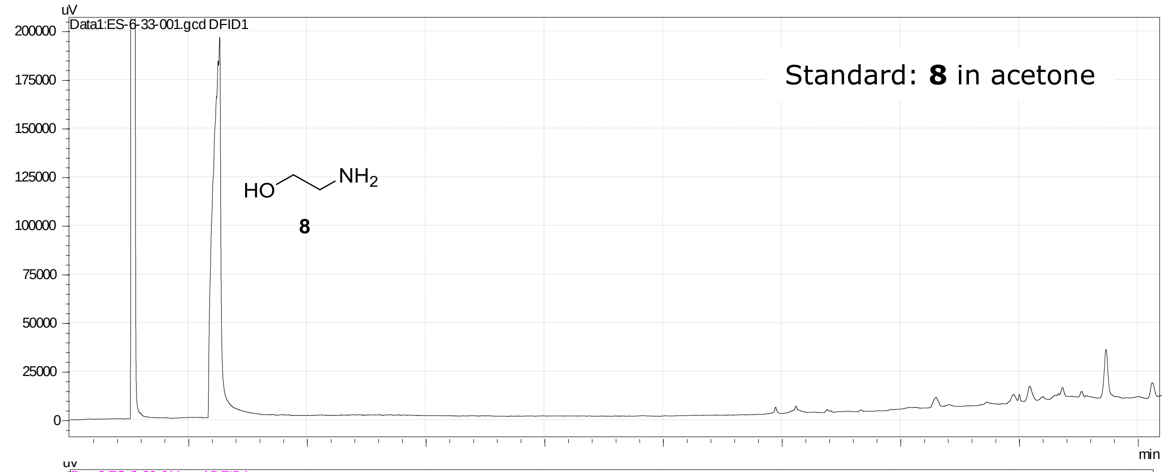

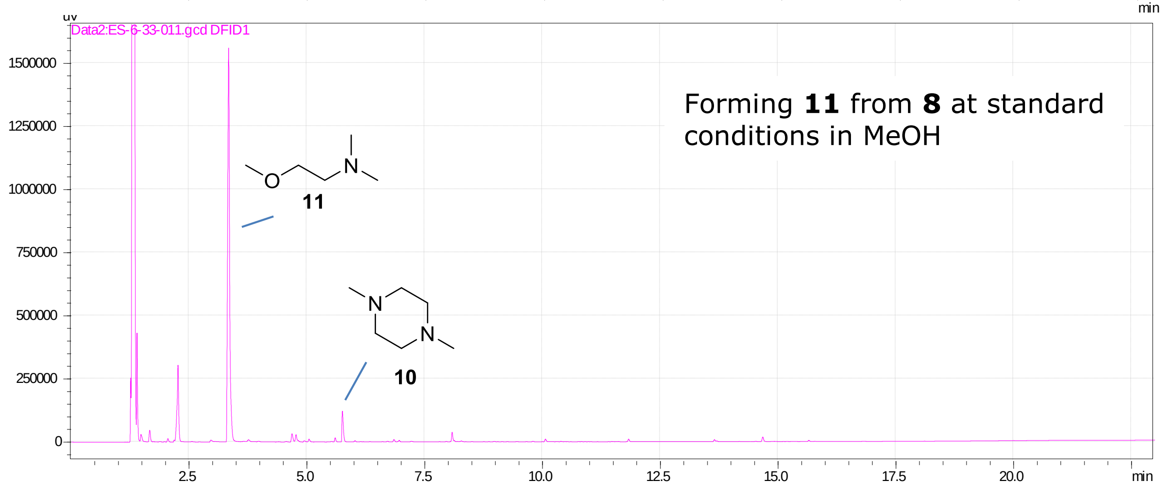

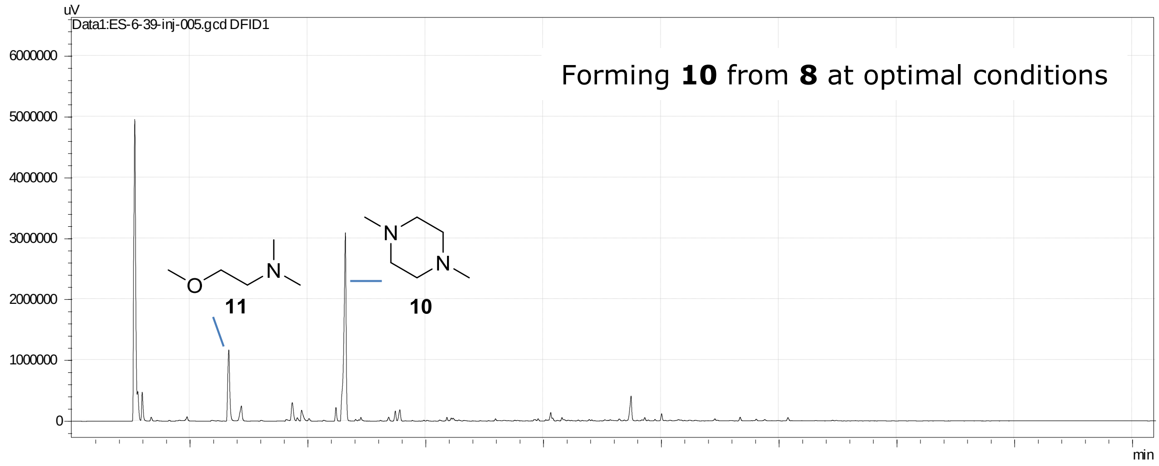

Formation of **13** and **14** from **12**.

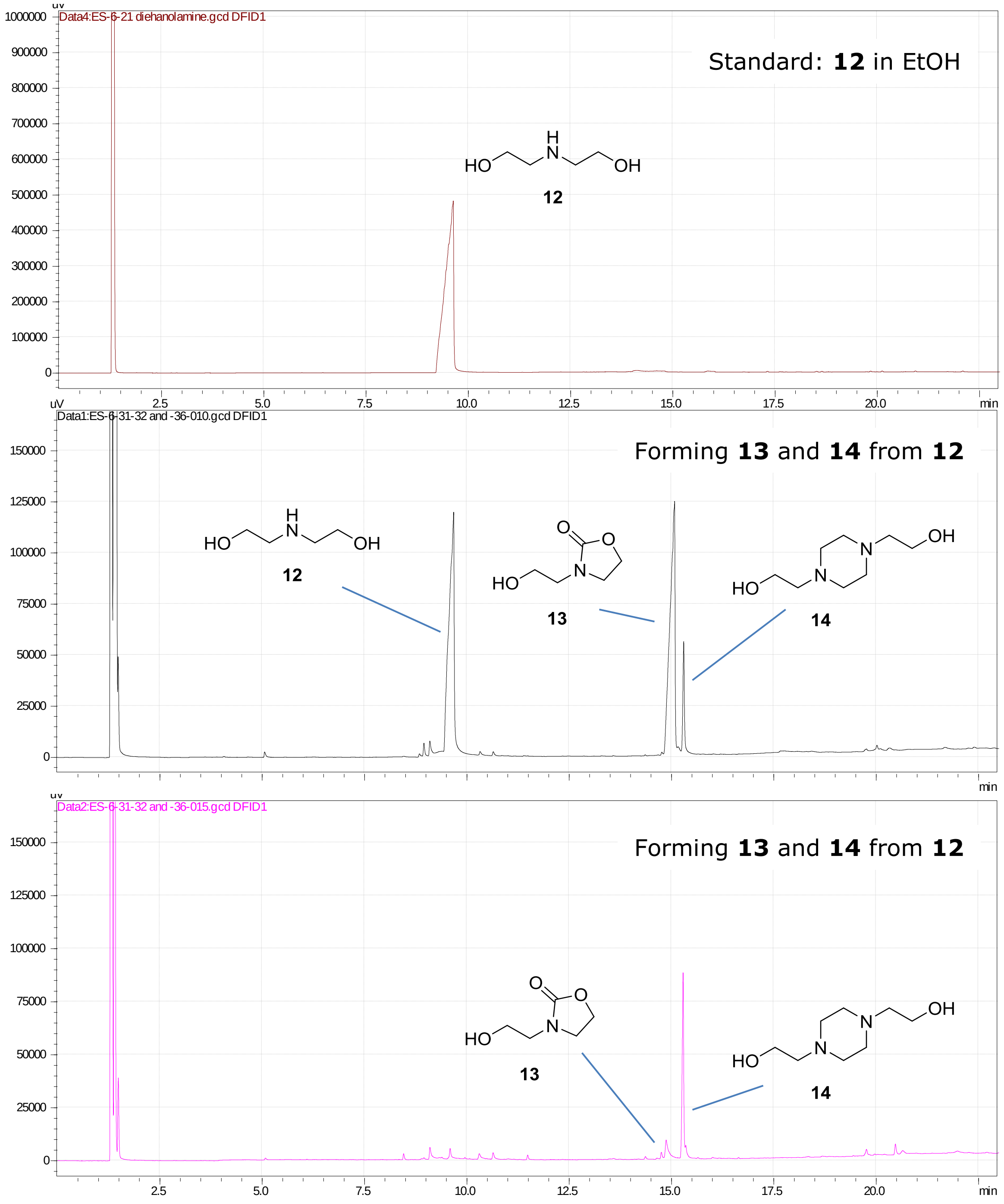

Formation of **9** from **12**.

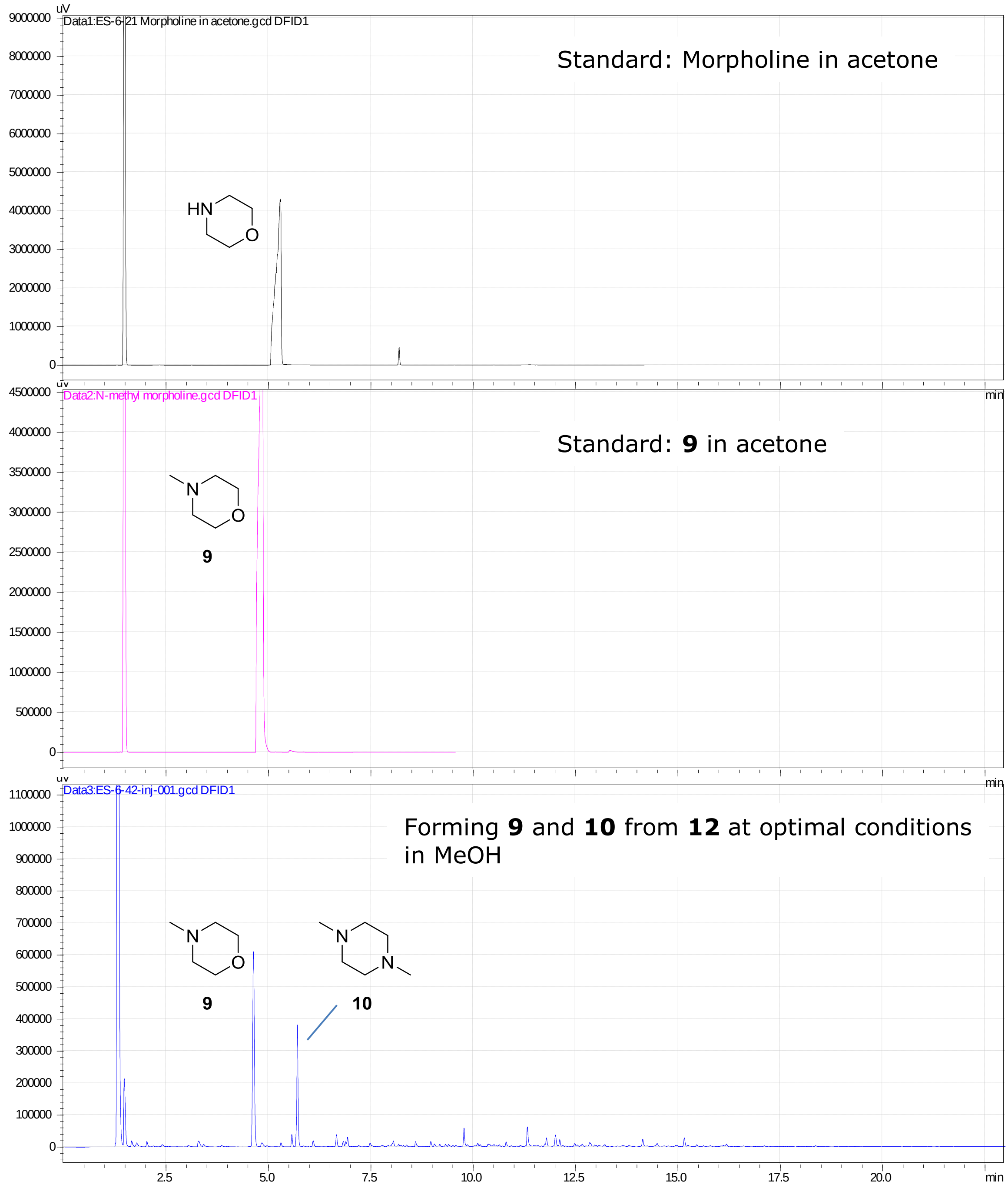

Formation of **12** from **15**.

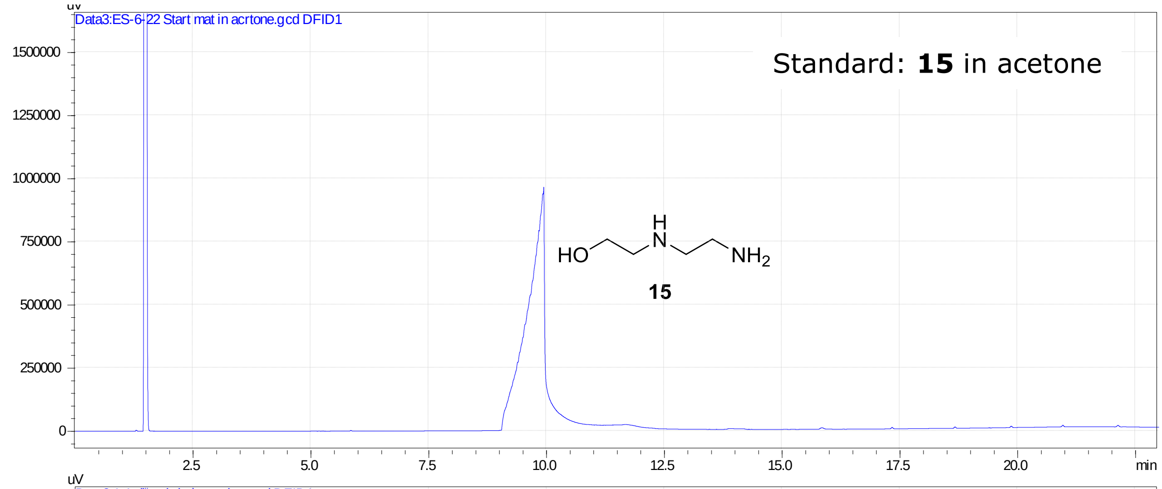

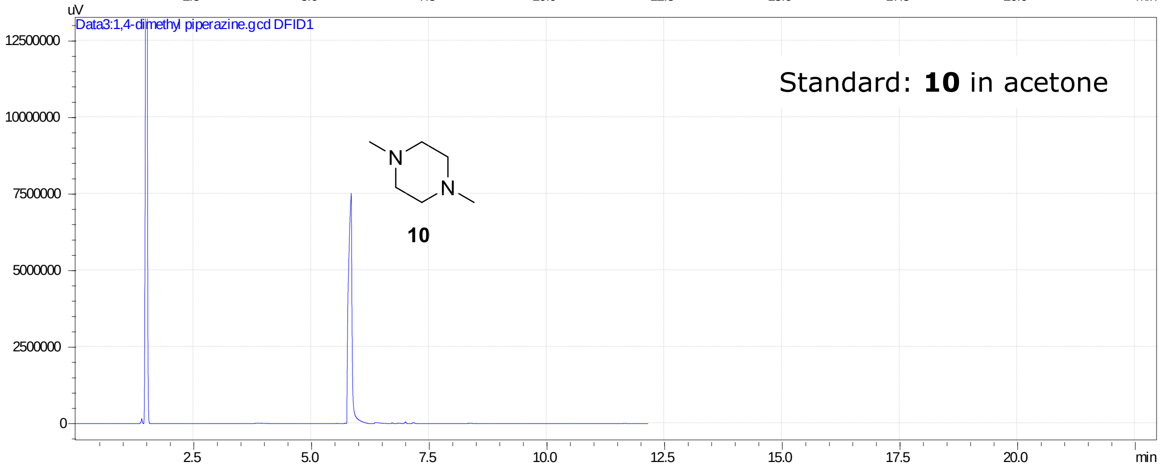

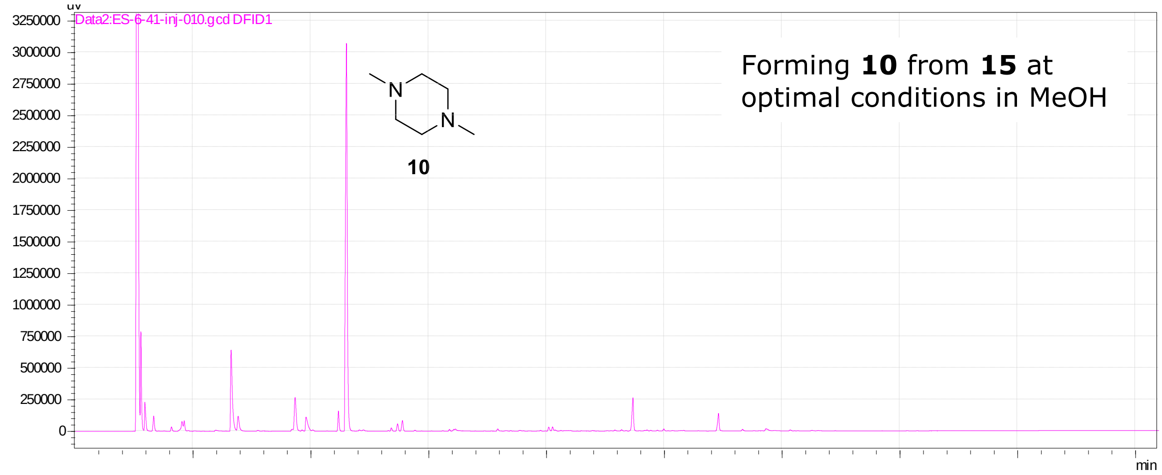

Formation of **16** from **15**.

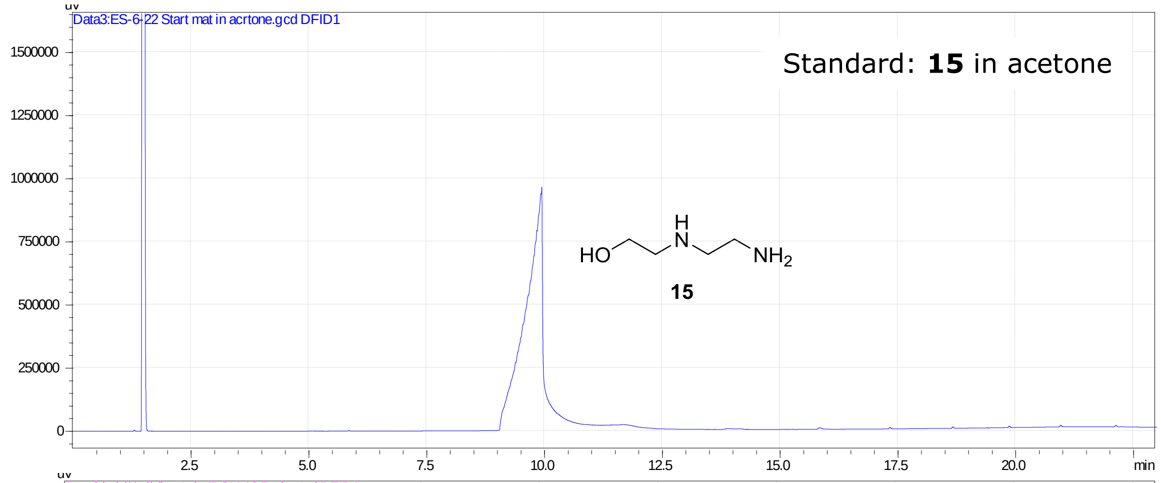

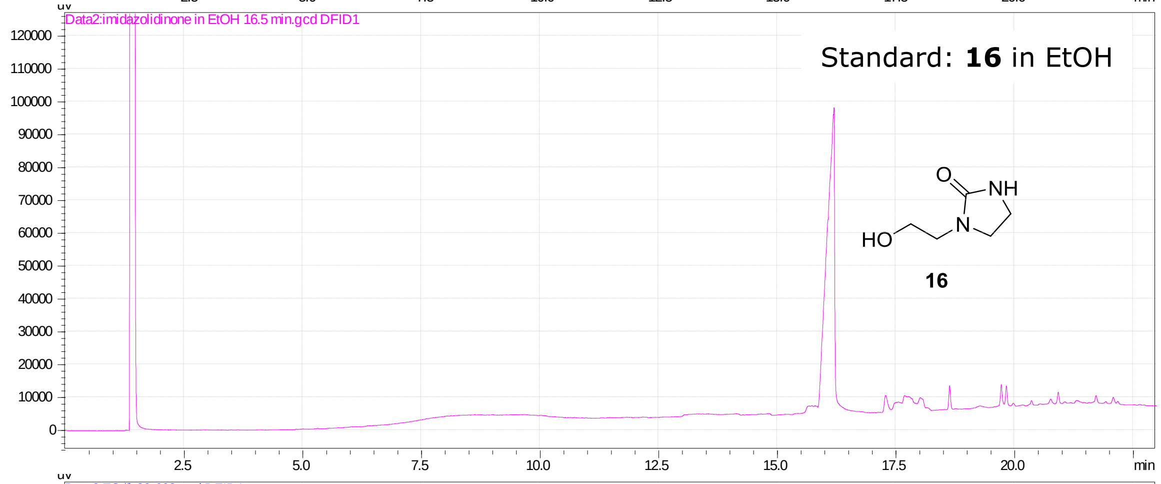

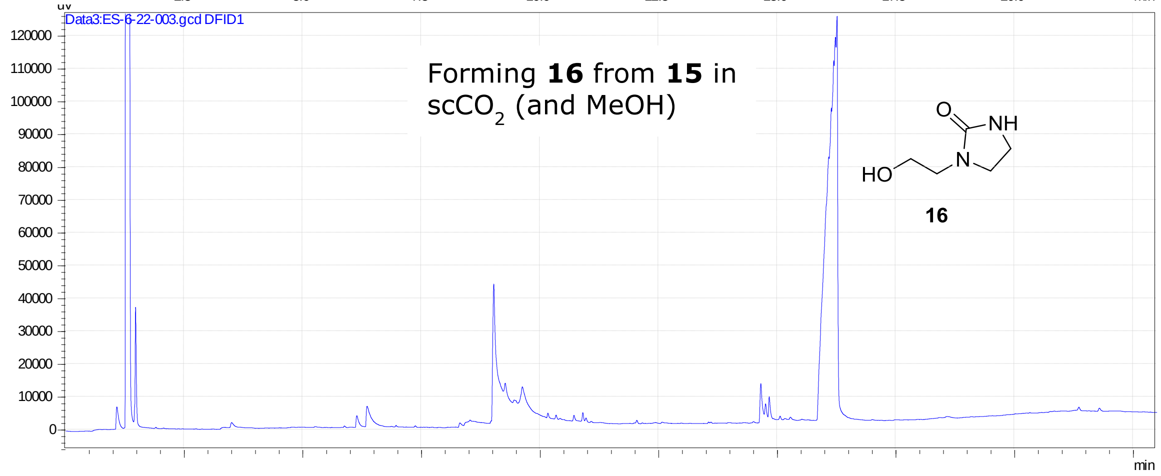

4. References

1. W. Huyer and A. Neumaier, *ACM Trans. Math. Software*, 2008, **35**, 1-25.
